# A multi-scalar perspective on health and urban housing: an umbrella review

**DOI:** 10.5334/bc.119

**Published:** 2021-08-31

**Authors:** Catalina Turcu, Melanie Crane, Emma Hutchinson, Simon Lloyd, Kristine Belesova, Paul Wilkinson, Mike Davies

**Affiliations:** The Bartlett Faculty of the Built Environment, University College London, London, UK; The Charles Perkins Centre, Sydney School of Public Health, The University of Sydney, Sydney, NSW, Australia; Public Health, Environments and Society, Faculty of Public Health and Policy, London School of Hygiene and Tropical Medicine, UK; Climate and Health Programme (CLIMA), Barcelona Institute for Global Health (ISGlobal), Barcelona, Spain; Public Health, Environments and Society, Faculty of Public Health and Policy, London School of Hygiene & Tropical Medicine, UK; Public Health, Environments and Society, Faculty of Public Health and Policy, London School of Hygiene & Tropical Medicine, UK; UCL Institute for Environmental Design and Engineering, Faculty of the Built Environment, University College London, London, UK

**Keywords:** building, built environment, environmental health, health, housing, mental health, metaanalysis, neighbourhood, urban

## Abstract

With more than half the world’s population living in cities, understanding how the built environment impacts human health at different urban scales is crucial. To be able to shape cities for health, an understanding is needed of planetary health impacts, which encompass the human health impacts of human-caused disruptions on the Earth’s natural ecosystems. This umbrella review maps health evidence across the spatial scales of the built environment (building; neighbourhood; and wider system, including city, regional and planetary levels), with a specific focus on urban housing. Systematic reviews published in English between January 2011 and December 2020 were searched across 20 databases, with 1176 articles identified and 124 articles screened for inclusion. Findings suggests that most evidence reports on health determinants at the neighbourhood level, such as greenspace, physical and socio-economic conditions, transport infrastructure and access to local services. Physical health outcomes are also primarily reported, with an emerging interest in mental health outcomes. There is little evidence on planetary health outcomes and significant gaps in the research literature are identified. Based on these findings, three potential directions are identified for future research.

## 1 Introduction

The influence of the urban built environment on human health is complex and multifaceted. Health is determined by local environmental exposure and behaviours of individuals, which are socially and spatially patterned (Marmot 2005). Material aspects of the built environment give rise to various social processes that may directly and indirectly shape the health of its residents (Rydin *et al*. 2012; Bai *et al*. 2016). This poses difficulties to both conceptualising the relation between health and the urban built environment, and when seeking to design and implement actions to protect and improve health.

The urban built environment may be broadly defined at three scales: building, neighbourhood, and the wider urban system encompassing processes that operate across the city and regional levels and extend as far as the planetary level. Addressing health at each spatial scale involves different types of planning and stakeholders, as well as governance frameworks. Hence, understanding the confluence of different scales is necessary to improve knowledge and actions in local urban environments (Ramaswami *et al*. 2016). A multi-scalar perspective of the local built environment provides a comprehensive view of how health interventions may be implemented more efficiently at a particular scale. Multiple health objectives and outcomes, as well as health risks, may be considered simultaneously across the spectrum of policymaking and systematically reflected in decision-making. This requires a thorough understanding of the interrelations between the different scales of the built environment in which a policy intervenes, and this reaches beyond the local level and across social and political boundaries. Ultimately, local-level health considerations should be linked to the health of the wider urban system, including planetary health, whereby environmental processes can both be disturbed by and threaten to disturb local living conditions and health (Rydin *et al*. 2012; Ramaswami *et al*. 2016; Whitmee *et al*. 2015).

Urban housing is a primary area for health intervention in the built environment. Current urbanisation estimates suggest that 68% of world’s population will live in cities by 2050. At the urban level, housing covers on average 70% of land-use, thus it is a substantial sector for policymaking (UN 2018). Traditionally, approaches to research supporting health-focused policy action in the housing sector have considered dwellings as atomised units of exposure (Thomson & Thomas 2015; Ige *et al*. 2019), although some recent work has begun to consider wider connections (Bird *et al*. 2018; Carlin *et al*. 2017; Núñez-González *et al*. 2020; Pérez *et al*. 2020). This paper aims to synthesise the current evidence on the built environment and health nexus, focusing on the urban housing sector as the entry point. The paper adopts the World Health Organization’s (WHO) (2018) relational definition of housing and includes the physical structure of the dwelling, its immediate environment and the community (WHO 2018). Many systematic reviews already exist of the health impact of interventions and/or risks within the house or built environment. However, addressing the broader issues for policy implementation and decision-making across scales requires a broad synthesis of the evidence. Umbrella reviews are useful for efficiently summarising and comparing the evidence, assessing such considerations of aspects of health across built scales and identifying research gaps (Aromataris & Munn 2020).

## 2 Methods

### 2.1 Literature Search Strategy

The umbrella review search strategy involved identifying peer-reviewed systematic reviews, published in the English language only, January 2011–December 2020. To ensure that both health and urban studies research is captured, 20 databases were included: MEDLINE, Academic Search Ultimate, CINAHL, Health Source Nursing/academic Edition, Family and Society Studies Worldwide, Environment Complete, Sociology Source Ultimate, GreenFILE, Engineering Source, Psychology and Behavioral Sciences Collection, Rehabilitation and Sports Medicine, Business Source Ultimate, Applied Science and Technology, Health Business elite, MasterFILE complete, Women’s studies international, Legal source, LGBTQ+ source and British Education Index, as well as Scopus.

The search strategy was based on the intersection (Boolean AND) of terms for housing and its area of influence at the local and built scale ((built environment OR slum* OR favella OR dwelling OR neighbourhood* OR house OR housing OR urban OR green space OR metropol* OR residen* OR accommodation OR informal settlement*) OR (((housing OR city OR cities OR urban OR municipal OR environment*) N4 (plan* OR strateg* OR design* OR infrastructure))) and terms for health (health N4 (outcome* OR effect* OR assessment* OR benefit* OR gain* OR cost* OR impact* OR hazard*)). Health terms were broad in order to identify a wide spectrum of health outcomes, including those relating to planetary health (including health effects of environmental pollution, climate change and water scarcity).

### 2.2 Eligibility Criteria

Housing as defined here (WHO 2018) included the dwelling and its immediate and wider surroundings, such as the neighbourhood services and facilities necessary for residential living, including green space, transportation, access to public services and facilities, and their connections to the city. Specific categories of housing such as student or elderly accommodation, residential care and shelters for the homeless were excluded. Systematic review articles published between January 2011 and December 2020 were eligible. Articles included meet the Cochrane Reviews criteria for systematic reviews: (1) clearly stated set of objectives with predefined eligibility criteria for included studies; (2) an explicit methodology; (3) a systematic search used to identify included studies; (4) an assessment of the validity of findings; and (5) a systematic presentation of results (Higgins *et al*. 2019).

### 2.3 Data Extraction

**Figure 1:**
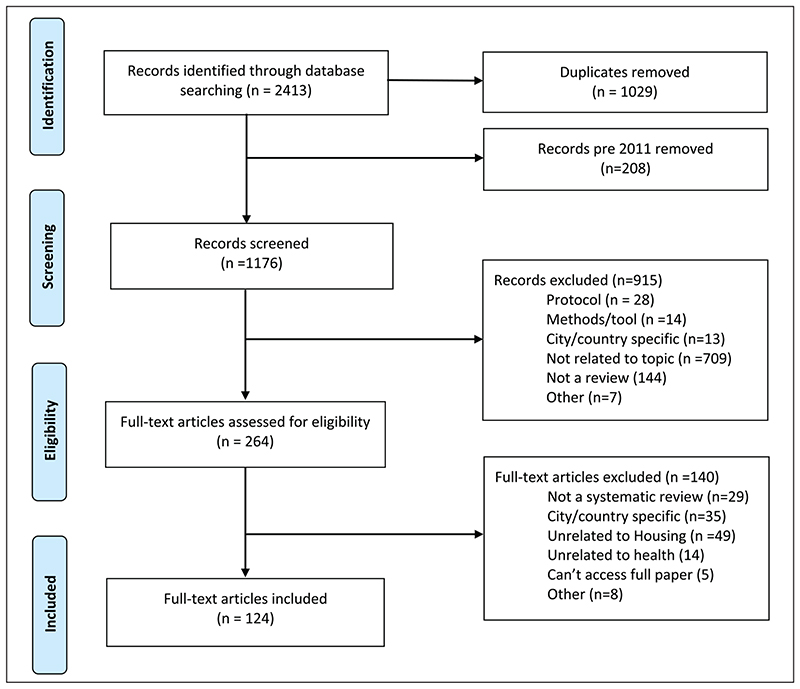
Study selection (PRISMA diagram).

From the initial number of 2413 articles found, 1029 duplicates and 208 studies published prior to January 2011 were removed, leaving 1176 for manual screening of titles and abstracts by the authors (Figure 1). Articles were rated by two reviewers independently. A total of 264 articles were selected for full text review, of which 124 were agreed by the reviewers to meet the inclusion criteria and thus included in this umbrella review. The reasons for exclusion are summarised in the PRISMA diagram shown in Figure 1.

### 2.4 Quality Assessment

The quality of included studies was assessed using the JBI manual for evidence synthesis of umbrella reviews (Aromataris & Munn 2020). The following aspects were recorded: the topic or intervention of each paper, the context or geographical location, reported outcomes, study type (*i*.*e*. experimental or observational studies) and a critical assessment of how the review evidence was synthesised. Information was also collated on (1) the built scale at which the study was focused (building, neighbourhood, urban system); (2) any mentioned of planetary outcomes or links to planetary outcomes; and (3) stakeholders (*i*.*e*. public/private stakeholders at a local, city, regional or national level) and governance mechanisms (*i*.*e*. policy, regulation, legislation, standard or guidance).

## 3 Results

### 3.1 Description of Study Quality and Characteristics

The 124 articles primarily included research from high-income countries (predominantly the UK, Europe and North America); few included studies from low-income countries (Alaazi & Aganah 2020; Henson *et al*. 2020; Jung *et al*. 2017; Katoto *et al*. 2019; Quansah *et al*. 2017; Shuvo *et al*. 2020; Turley *et al*. 2013), and these were mainly in relation to urban slum interventions or impacts. The types of studies included in each review were diverse, but mainly collated evidence from cross-sectional population surveys. The majority of reviews assessed the quality of evidence in terms of potential publication bias and used criteria for appraising studies for inclusion. While the majority commented on the poor quality and potential bias of included papers, quality was not always stated. Few reported research from randomised controlled trials or quasi-experimental studies, and yet the quality of studies was generally reported by authors as medium quality. Study designs were generally broad, inclusive of qualitative and non-experimental evidence. The built environment was often broadly defined, although a few studies incorporated geographical information system (GIS) indicators when assessing outcomes at the neighbourhood or city scale (Gong *et al*. 2016; Ma *et al*. 2020; Malambo *et al*. 2016; McCrorie *et al*. 2014; Nordbø *et al*. 2018).

**Figure 2:**
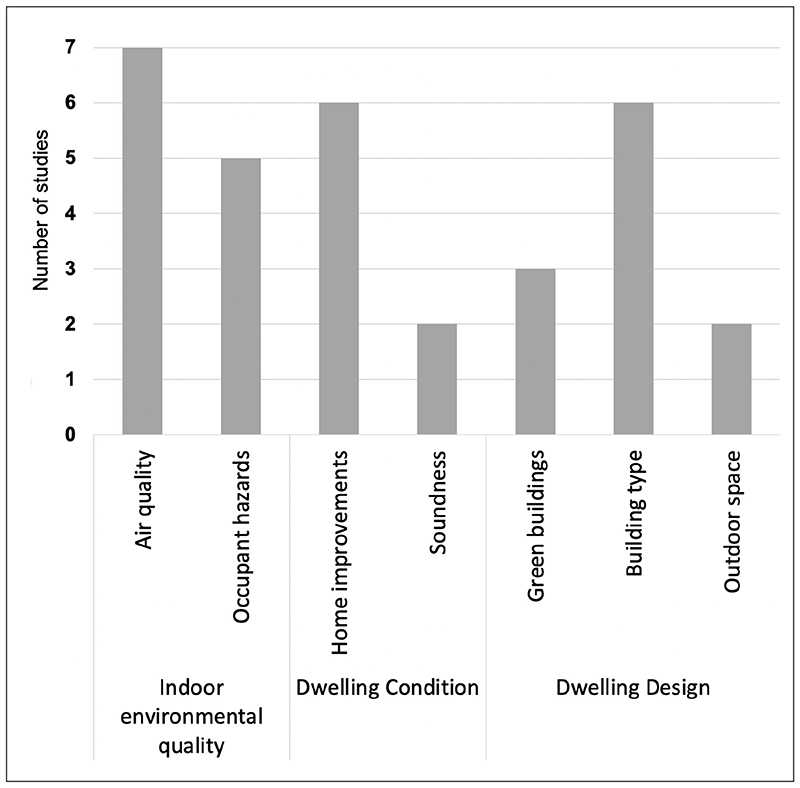
Number of studies looking at the DWELLING scale (31 systematic reviews).

**Figure 3:**
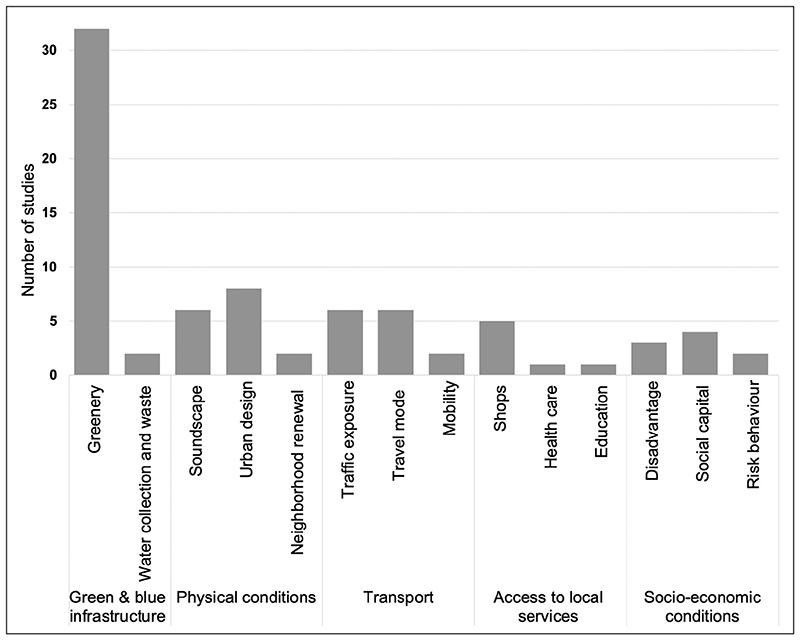
Number of studies looking at the NEIGHBOURHOOD scale (80 systematic reviews).

**Figure 4:**
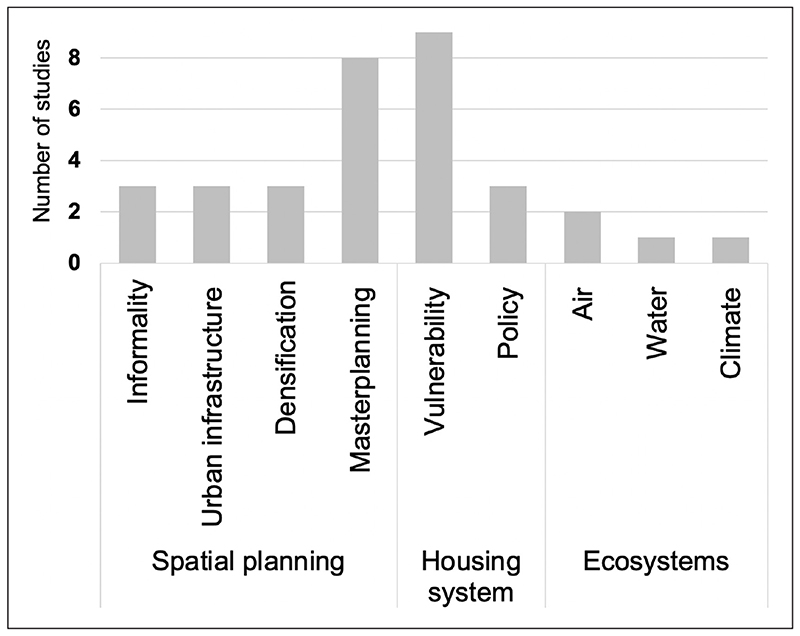
Number of studies identified at the wider URBAN SYSTEM scale (32 systematic reviews).

The majority of studies focused on the neighbourhood scale (*n* = 80), followed by the dwelling (*n* = 31) and urban system (*n* = 32) level. Some studies addressed multiple scales, but few identified these from a scale perspective (Levy-Storms *et al*. 2018). Most focused on non-communicable disease and physical health, especially at the neighbourhood level; mental health was mostly examined at the neighbourhood level. At the dwelling level, physical and mental health outcomes featured with a greater focus on cardiorespiratory outcomes related to indoor air pollution and other hazards. Many studies focused on the general population, but some were of more specific populations, including children (*n* = 20), older adults (*n* = 9), mothers and pregnant women (*n* = 3), the socially disadvantaged (*n* = 5) or people with a specific chronic disease (*i.e*. HIV, diabetes, asthma) (*n* = 4). A narrative synthesis of the findings follows below, structured under the three broad levels of housing at scale: building, neighbourhood and urban system. Figures 2–4 show the number of studies identified at each spatial level.

### 3.2 Building Determinants of Health (31 Studies)

A total of 31 (25%) of the systematic reviews reported factors that affect health at the building or dwelling level under three broad areas: (1) *indoor environmental quality* (12 studies) such as *air quality* (*e*.*g*. indoor pollution from location and construction materials) and *occupant hazards* (*e*.*g*. burning fuel, cooking, heating); (2) *dwelling condition* (eight studies) such as *home improvements* (*e*.*g*. energy retrofits, bathroom/kitchens) and *soundness* (*e*.*g*. collapse and fire hazard); and (2) *dwelling design* (11 studies), including *green buildings* (*e*.*g*. green walls, green design, green standards), *building type* (*e*.*g*. high-rise, apartment, cohousing, shared facilities) and *outdoor space* (*e*.*g*. gardens) (Table 1; and see Table S1 in the supplemental data online).

#### 3.2.1 Indoor environmental quality

A total of 12 systematic reviews were included in this category looking at *air quality* (seven studies) and *occupant hazards* (five studies). The *air quality* subcategory included studies of outdoor air pollution in the vicinity of the dwelling (*e*.*g*. originating from such sources as a nearby traffic) (Vardoulakis *et al*. 2020), its infiltration into the dwelling, and the associated risks for the cardiovascular and respiratory health of the residents, including asthmatic and allergic exacerbations (Tagiyeva & Sheikh 2014; Van Boven *et al*. 2020). The extent to which outdoor air pollution enters the dwelling is related to the building structure, its fabric, operation and ventilation/air purification technologies (Van Boven *et al*. 2020). This subcategory also included studies of radon, lead, volatile and semi-volatile organic compounds (VOCs, SVOCs) (Ajrouche *et al*. 2017; Naldzhiev *et al*. 2020; Nussbaumer-Streit *et al*. 2020) as well as of biological agents (Sharpe *et al*. 2015). The *occupant hazards* subcategory looked at wood and biomass burning for heating and cooking as well as cookstove efficiency, which are important determinants of poor household air quality and impact on a wide array of health endpoints (Bailey *et al*. 2019; Quansah *et al*. 2017); low indoor temperatures may also direct effect blood pressure, blood clotting and other pathophysiological changes (Jevons *et al*. 2016). Three studies addressed issue of environmental equity in relation to exposure to emissions from domestic wood fuel burning (Bailey *et al*. 2019), air pollution impacts from cook stove and biomass burning (Katoto *et al*. 2019), and thermal control in homes of the elderly (Jevons *et al*. 2016).

#### 3.2.2 Dwelling condition

Eight studies were grouped in this category under *home improvements* (six studies) and *soundness* (two studies). Of the six studies on *home improvements*, three focused on energy efficiency retrofitting (Ige *et al*. 2019; Pega & Wilson 2016; Naldzhiev *et al*. 2020; Bailey *et al*. 2019; Fenwick *et al*. 2013; Jevons *et al*. 2016), with one including interventions to reduce falls (Carnemolla & Bridge 2020). The two *soundness* studies reported on injuries from fire hazards (Senthilkumaran *et al*. 2019; Bailey *et al*. 2019; Fenwick *et al*. 2013; Jevons *et al*. 2016). Of all studies in this category, only those relating to home improvements reported on multiple health impacts; in addition to physical health, three studies looked at mental health (Garin *et al*. 2014; Ige *et al*. 2019; Fenwick *et al*. 2013) and one focused on the elderly (Garin *et al*. 2014).

#### 3.2.3 Dwelling design

A total of 11 studies were identified under this category, further subcategorised into three groups: *green buildings* (three studies) looking at green walls, green design and green standards; *building type* (six studies) to do with high-rise, type of dwelling, shared facilities, *etc.;* and *outdoor space* (two studies). The green *buildings studies* assessed a range of health outcomes, including communicable disease (associated with water systems, vector-borne disease), allergens and air quality (Allen *et al*. 2015; Houghton & Castillo-Salgado 2017, 2019). The *building type studies reported on physical space and mental health and wellbeing* (Barros *et al*. 2019; Garin *et al*. 2014), the impact of accessible design on falls and mental health (Cho *et al*. 2016), and how design can minimise risks of injury, particularly later in life (Garin *et al*. 2014); sedentary lifestyles associated with apartment or duplex living (Chastin *et al*. 2015); physical and mental health, and wellbeing (Carrere *et al*. 2020); and shared sanitation’s impact on communicable disease (Heijnen *et al*. 2014). Two studies looked at health impacts associated with a dwelling’s *outdoor space*, including one on the physical activity benefits of gardens and outdoor equipment for children (Carlin *et al*. 2017), and one on stress and time spent gardening (Kondo *et al*. 2018b).

#### 3.2.4 Summary of building results

All systematic reviews in this category reported on physical health non-communicable outcomes associated with respiratory health (derived from exposure to allergens and air pollution) such as asthma, allergic, cardiovascular, blood pressure or thermoregulation conditions; and safety/physical injury from specific housing types (*e*.*g*. duplex living). Only four reviews reported on communicable disease risks associated with water systems and shared sanitation as determinants for vector-borne disease. Compared with physical health, mental health outcomes were less reported (six studies) and in relation to a dwelling condition (*e*.*g*. thermal comfort) and design (*e*.*g*. type of building). Some reviews focused on vulnerable groups such as children and the elderly (indoor environmental quality and dwelling condition studies), the elderly (dwelling condition) and the disabled (dwelling design).

There was limited consideration of planetary health impacts (six studies). Studies reported on associated carbon emissions from traffic (Vardoulakis *et al*. 2020) and from compensatory ventilation to address indoor air pollution and emissions from particular types of thermal insulation (Naldzhiev *et al*. 2020); greenhouse gas mitigation, flood risk management and ecological impacts for wildlife (Allen *et al*. 2015; Houghton & Castillo-Salgado 2017, 2019); and energy efficiency and efficient use of resources arising from high-density living (Barros *et al*. 2019).

### 3.3 Neighbourhood Determinants of Health (80 Studies)

A total of 80 studies (64.5%) reported on various aspects of the neighbourhood, which are broadly defined here as an urban area made of residential and other buildings, as well as the supporting infrastructure for everyday living and its resident community. These were organised under five broad categories: (1) *green and blue infrastructure* (34 studies), looking at *greenery, water collection and waste;* (2) *physical conditions* (16 studies), reporting on the effects of *soundscape (i.e*. noise from traffic), *urban* design (*i.e*. street layout, lighting, walkability), and neighbourhood *renewal;* (3) *transport* (14 studies), including *traffic exposure, travel mode* and *mobility* aspects; (4) *access to local services* (seven studies), such as *shops, healthcare* and *education;* and (5) *socio-economic conditions* (nine studies), reporting on effects of *disadvantage, social capital* and *crime* on health outcomes (Table 2; and see Table S2 in the supplemental data online).

#### 3.3.1 Green and blue infrastructure

A total of 34 studies were concerned with the relationship between *green and blue infrastructure* and health, grouped under *greenery* (32 studies) regarding various green aspects of neighbourhoods, including parks, greenspace, vegetation, trees, *etc*.; and *water collection and waste* (two studies) reporting on sewerage systems and water-borne pathogens (Jung *et al*. 2017) and pathogens in floodwater and respiratory disease (Ishaq *et al*. 2020).

Of the *greenery* studies, 12 considered the relationship with physical health impacts only, mainly in the area of respiratory and cardiovascular disease or general health impacts (Browning & Lee 2017; Twohig-Bennett & Jones 2018; Parker & de Baro 2019; Rugel & Brauer 2020); and 15 studies also reported on mental health outcomes. Several reviews focused on the health of particular population groups including older adults and mortality (Rugel & Brauer 2020; Yuan *et al*. 2020; Rojas-Rueda *et al*. 2019); children and asthma (Hartley *et al*. 2020); pregnancy outcomes (Akaraci *et al*. 2020; Lee *et al*. 2020c); and early childhood (Islam *et al*. 2020). Three studies specifically focused on the benefits of physical activity for health more generally (Macmillan *et al*. 2018), children and adults with disabilities (Saitta *et al*. 2019), and older adults (Chastin *et al*. 2015). Five studies assessed various mental health outcomes, including stress and anxiety more generally (Calogiuri & Chroni 2014; Felappi *et al*. 2020; Gascon *et al*. 2017; Kondo *et al*. 2018a; Chastin *et al*. 2015), and in children and adolescents (Vanaken & Danckaerts 2018). One study assessed the mediating effects of vegetation on the relationship between stress and noise (Dzhambov & Dimitrova 2018). Six studies reported other types of outcomes in addition to health including benefits to the economy and society (Venkataramanan *et al*. 2019), social capital (Venkataramanan *et al*. 2019), cognitive development in childhood and cognitive function in adulthood (de Keijzer *et al*. 2016), and cognitive function in children, adults and the elderly (de Keijzer *et al*. 2020); environmental and health inequalities (Schüle *et al*. 2019); and food security and nutrition outcomes (Audate *et al*. 2019). The relationship between noise and green space was investigated in one study (Dzhambov & Dimitrova 2018).

#### 3.3.2 Physical conditions

A total of 16 studies were grouped under this category considering *soundscapes* from road traffic mainly (six studies), aspects of *urban design* (eight studies) and *neighbourhood renewal* (two studies). Of these, six reviews examined the relationship between *soundscape* and health impacts, including noise associations with hypertension (Dzhambov & Dimitrova 2018; Van Kempen & Babisch 2012) and myocardial infarction (Khosravipour & Khanlari 2020); pathways to health from noise (Peris & Fenech 2020); noise levels, stress and self-reported general health (Aletta *et al*. 2018); and excessive noise and disability in later life (Garin *et al*. 2014). A further eight studies looked at different aspects of *urban design:* half of these were studies of physical activity and walkability, specifically looking at disabled people (Eisenberg *et al*. 2017), successful ageing (Garin *et al*. 2014) and the elderly (Moran *et al*. 2014; Chastin *et al*. 2015); and three studies looked at physical health impacts associated with obesity-related outcomes such as type 2 diabetes and hypertension (Chandrabose *et al*. 2019; Malambo *et al*. 2016; Schüle & Bolte 2015), one of which also considered mental health impacts (Schüle & Bolte 2015) and another cardiometabolic risk (Leal & Chaix 2011). Two studies reported on health outcomes associated with *neighbourhood renewal:* they focused on health impacts of housing regeneration schemes and socio-economic determinants of health (Thomson & Thomas 2015), and on impacts on mental and wellbeing from improvements in neighbourhood infrastructure such as access to transport and street greening (Moore *et al*. 2018).

#### 3.3.3 Transport

This category encompasses 14 studies grouped in three subcategories: *traffic exposure* (six studies), *travel mode* (six studies) and *mobility* (two studies). Studies looking at *traffic exposure* reported on cardiovascular outcomes (Malambo *et al*. 2016) in addition to mortality and other physical health impacts (Rugel & Brauer 2020); lung cancer (Hamra *et al*. 2015); and children and lung function (Boothe *et al*. 2014), leukaemia (Barone-Adesi *et al*. 2015) and obesity (Audrey & Batista-Ferrer 2015). Six studies reported on impacts of *travel mode* on health. Three studies were specifically concerned with cycling interventions, two looked at the potential for general public health impacts (Stewart *et al*. 2015; Stankov *et al*. 2020), and one at physical activity and diet-related health outcomes (Macmillan *et al*. 2018). One study considered public transport and weight-related health outcomes (Patterson *et al*. 2019). The remaining two studies looked at transport costs and health impacts (Möller *et al*. 2020), and mode of travel and health impacts in children from lower socio-economic groups (Ma *et al*. 2020). Two reviews looked at transport *mobility* issues such as barriers to transport arising from congenital cardiovascular conditions in children and adults (Davey *et al*. 2020), and transport mobility restrictions and health impacts including premature mortality in older adults (Rosso *et al*. 2011).

#### 3.3.4 Access to local services

Seven studies were grouped in this category. Five studies focused specifically on access to and availability of local shops, with four looking at grocery shops and local food outlets such as supermarkets, farmers’ markets, restaurants and community kitchens (Abeykoon *et al*. 2017; Macmillan *et al*. 2018; Malambo *et al*. 2016; Iacovou *et al*. 2013), and one at retail more generally (Garin *et al*. 2014). In terms of health impacts, one study looked at access to healthcare and congenital heart disease in children and adults (Davey *et al*. 2020), and another reported on the health of children from lower socio-economic groups and exposure to traffic from their route to school (Ma *et al*. 2020). Two studies considered body mass index (BMI)-related outcomes in relation to grocery stores and supermarkets (Abeykoon *et al*. 2017; Malambo *et al*. 2016), one of which also considered blood pressure, diabetes mellitus and metabolic syndrome associated with fast-food restaurants (Malambo *et al*. 2016). Two studies reported on mental health outcomes: one looked at depression in the elderly and retail availability in the neighbourhood (Garin *et al*. 2014); and another looked at self-reported health and psychological health and grocery shops (Abeykoon *et al*. 2017). One study linked community kitchens with wellbeing benefits such as social engagement and community cohesion (Iacovou *et al*. 2013).

#### 3.3.5 Socio-economic conditions

The socio-economic characteristics of neighbourhoods’ impact health as they are underlying factors of: disadvantage and competition for scarce resources among neighbours; trust, social capital and collective action which can overcome challenges; and ‘contagious’ or ‘epidemic’ behaviours that makes neighbours engage in similar behaviours (Smelser & Baltes 2001). Nine studies were identified in this category reporting on *disadvantage* (three studies), *social capital* (four studies) and *risky behaviours* (two studies).

While only two studies reported on general physical health outcomes related to gentrification (Bhavsar *et al*. 2020) and adolescent pregnancy (Smelser & Baltes 2001), the overall focus in this category and touched upon by all studies was on mental health conditions (*e*.*g*. depression, anxiety, self-reported health, *etc.*) (five studies), health-risk behaviour (*e*.*g*. smoking, physical inactivity and early sex initiation) (two studies), and wellbeing (*e*.*g*. social health, loneliness) (four studies) outcomes. Moreover, many studies reported health impacts on vulnerable groups such children (Bhavsar *et al*. 2020; Vyncke *et al*. 2013; Decker *et al*. 2018), ethnic minority populations (Bécares *et al*. 2018; Bhavsar *et al*. 2020) and lower socio-economic groups (Bhavsar *et al*. 2020). One paper reported on the health impacts on all these three groups and also referred to planetary health outcomes in relation to environmental equity aspects of ‘green gentrification’ which can result in displacing vulnerable residents and augment their need for more emergency room or mental health visits as well as their food insecurity (Bhavsar *et al*. 2020).

The impact of *disadvantage* on health was reported in relation to neighbourhood deprivation and health-risk behaviour such as smoking and physical inactivity (Algren *et al*. 2015); ethic segregation and mental health outcomes such as depression, anxiety, suicidality and suicide, psychotic experiences, and schizophreniform/psychotic disorders (Smelser & Baltes 2001); and gentrification and self-reported health, physical and mental health outcomes and health-related behaviour, with a specific focus on negative health outcomes for ethnic groups, children and displaced residents (Bhavsar *et al*. 2020). Four studies reported on *social capital*-related health outcomes looking at the amount of social capital in the neighbourhood and health outcomes in children and adolescents (Vyncke *et al*. 2013); the benefits of spending time with others and mental health, quality of life and social health (Lee *et al*. 2020b); social cognition from leisure-time and health outcomes (Rhodes *et al*. 2018); and community life and healthy weight and depression (Pérez *et al*. 2020). Two studies reporting on *risky behaviours* looked at unsafe local environments, early sexual initiation and adolescent pregnancy (Decker *et al*. 2018), fear of crime and mental health (Lorenc *et al*. 2013).

#### 3.3.6 Summary of neighbourhood results

Across all reviews in this group, three overall findings were apparent: (1) the *green and blue infrastructure* category received the most attention to date (34 studies); (2) physical health outcomes (respiratory and cardiovascular) were predominantly reported; however, mental health outcomes were also reported by 27 studies, especially in relation to greenspace (15 studies) and neighbourhood socio-economic conditions (nine studies)—interestingly, no transport study reported mental health outcomes; and (3) in contrast to the building level/group, nine studies reported on the socio-economic determinants of health and discussed these in relation to various vulnerable groups including children (15 studies), elderly (10 studies), disabled people (three studies), women (three studies), low income (two studies) and ethnic minority groups (two studies); the intersectionality of health outcomes was also considered in three studies reporting on adverse health outcomes on low income and children or women.

One-fifth of studies (*n* = 16) reported on planetary health aspects, predominantly under the *greenery* category (nine studies). Reported aspects included biodiversity (Lai *et al*. 2019), (cultural) ecosystem services (Zhang *et al*. 2017), conflicts between wildlife and human needs (Felappi *et al*. 2020), environmental resources (Schüle *et al*. 2019), environmental benefits (Parker & de Baro 2019) and air pollution (Lee *et al*. 2020c), sustainability-related aspects such as flooding (Ishaq *et al*. 2020; Venkataramanan *et al*. 2019) and the United Nations’ Sustainable Development Goals (SDGs) (Vanaken & Danckaerts 2018); mediating effects of weather and environmental conditions on physical activity in older adults (Moran *et al*. 2014); carbon emissions from road traffic (Barone-Adesi *et al*. 2015; Hamra *et al*. 2015; Rugel & Brauer 2020); and air pollution from transport (Möller *et al*. 2020; Ma *et al*. 2020).

### 3.4 Urban System Determinants of Health (32 Studies)

Local built environments are part of the wider urban system which extends beyond buildings and neighbourhoods to the whole city, immediate but also distant built or unbuilt hinterlands of regions, nations and, ultimately, to the planet. These are parts of the urban system connected by complex relations and feedback loops, which in turn can influence outcomes at the local level. In the housing sector, this occurs through, but it is not limited to, (1) planned action of housing at scale via *spatial planning;* (2) the unplanned, albeit regulated, interactions of agents and institutions seeking and providing housing represented by the *housing system;* and (3) via the consequential desired and undesired impacts on natural *ecosystems*. A total of 32 systematic reviews (25.8%) reported on urban system determinants of health categorised as *spatial planning* (17 studies); *housing system* (11 studies): and *ecosystems* (four studies) (Table 3; and see Table S3 in the supplemental data online).

#### 3.4.1 Spatial planning

A total of 17 studies were identified in this category reporting on health outcomes and *informality* (three studies), *urban infrastructure* (three studies), *type of development* (three studies) and *master planning* (eight studies). The three studies reporting on *informality* assessed the health impacts of strategies to improve the infrastructure, conditions and land tenure of slums, including communicable and non-communicable disease prevention, the risk of injury from chemical and biological hazards, as well as social impacts such as quality of life, education and employment (Alaazi & Aganah 2020; Turley *et al*. 2013; Henson *et al*. 2020). Three studies looked at *urban infrastructure*: one study assessed urban drinking water and gastroenteritis risk (Beaudeau 2018); one study reported on the structural soundness of the city in the face of earthquakes and subsequent building collapses (Doocy *et al*. 2013); and one study looked at urban exposure to overhead powerlines (Habash *et al*. 2019). *Type of development* effects were reported in three studies in relation to walkability, physical activity behaviours and obesogenic health impacts (Berghauser Pont *et al*. 2020; Chandrabose *et al*. 2019; Cyril *et al*. 2013). Eight studies focused on aspects of *master planning* at the city level and health outcomes such as morbidity and mortality related to non-communicable disease risks (*i.e*. physical inactivity), injury and mental health. More specifically, these studies focused on nature-based approaches (Kabisch *et al*. 2017), age-friendly infrastructure for the elderly or children (Sánchez-González *et al*. 2020; Nordbø *et al*. 2018), and smart city technologies (Rocha *et al*. 2019). Strategic planning and smart growth approaches were also included (McCrorie *et al*. 2014; Durand *et al*. 2011; Gong *et al*. 2016; Salgado *et al*. 2020).

#### 3.4.2 Housing system

Housing system studies (*n* = 11) were categorised into two categories: *vulnerability* (eight studies) and *policy* (three studies). In the *vulnerability* subcategory, three studies looked at how foreclosure, either directly experienced or general risk in the neighbourhood, negatively affected physical and mental health, as well as health-relevant behaviours (including substance misuse and violence) (Downing 2016; Tsai 2015; Vásquez-Vera *et al*. 2017); one study looked at how combinations of tenure precarity and poor physical characteristics of dwellings may combine to influence mental health (Singh *et al*. 2019); one study assessed how permanent supportive social housing may benefit both health (*e*.*g*. mental health, hospital admissions) and health-supporting social conditions (*e*.*g*. employment and income) (Aubry *et al*. 2020); and three studies looked at vulnerability by specifically focusing on a population subgroup (people living with HIV) (Aidala *et al*. 2016), a risk type (cold weather) (Tanner *et al*. 2013), and an outcome (congenital heart disease) (Davey *et al*. 2020). Of the three studies classified in the *policy* subcategory, two looked at how a range of material housing changes and support contributed to changes in the risk of obesity (Tseng *et al*. 2018) and diabetes (Barnard *et al*. 2015). A third study considered strategies that combined material (*e*.*g*. built infrastructure) and social (*e*.*g*. community networking and empowerment) aspects to create healthy environments (*e*.*g*. availability of healthy food; encouragement of physical activity) that would in turn improve community health (Chaparro *et al*. 2020).

#### 3.4.3 Natural ecosystems

Four studies reported on natural *ecosystems* such as *air*, *water* and *climate*. Two studies considered air pollution and its effects on cardiorespiratory outcomes in people of different age groups in Sub-Saharan Africa (Katoto *et al*. 2019), and the health and health equity benefits of interventions aiming to reduce air pollution levels (Benmarhnia *et al*. 2014). One study assessed the mental health benefits of wider natural ecosystems for residential areas (Gascon *et al*. 2017), and one paper looked at microclimate influences on urban heat islands and the resulting impacts of mortality and cardiorespiratory morbidity (Schinasi *et al*. 2018).

#### 3.4.4 Summary of urban system results

More than half (17 studies) of all studies in this group focused on the impact of *spatial planning* on both health exposure and health interventions. Physical health impacts were discussed in relation to both communicable and non-communicable diseases, more specifically general health outcomes, mortality and morbidity, walkability and physical activity outcomes, drinking water quality and communicable disease, resilience to natural disasters, cancer, reproductive health, obesity and cardiorespiratory. Mental health impacts were discussed in relation to *informality* and *master planning* aspects; *housing system’s* vulnerability (mainly measures of housing insecurity) and policies (material change and material and social support) aspects, and the natural *ecosystem*. There was some evidence reporting on the health of particular demographic groups such as the elderly and children (under *spatial planning*), and vulnerable groups including those with underlying health conditions (HIV, heart disease) and especially those with insecure housing tenure (under *housing system*).

Surprisingly, less than one-sixth of studies (*n* = 5) discussed planetary health outcomes. These reported on the impacts of rapid urbanisation on the environment and implications for the SDGs (Henson *et al*. 2020; Chandrabose *et al*. 2019); and climate change impacts of air pollution (Benmarhnia *et al*. 2014; Katoto *et al*. 2019) and microclimates (Schinasi *et al*. 2018).

## 4 Discussion

This paper provides an overview of the last decade’s evidence on health and urban housing from a spatial scale perspective. Most of the systematic review evidence (64.5%) reviewed by this paper focused on the *neighbourhood* level of the local built environment, while the *building* and *urban system* levels accounted for the rest in equal shares. Across the three scales, however, five out of 11 categories of built environment determinants of health have received most attention to date: *green and blue structure* (32/124); *spatial planning* (17/124), neighbourhood’s *physical conditions* (16/124) and *transport* (14/124), and a dwelling’s *indoor environmental quality* (12/124). The number of reviews focusing on health impacts at the building level was less than anticipated; this can be explained by the fact that existing evidence is not yet published in English and/or summarised by systematic reviews during the period 2011–20.

Three overall observations can be made across all scales and all studies. Physical health outcomes remained predominantly reported by systematic reviews, primarily in relation to non-communicable diseases in high-income settings. This may be because the majority of reviews focused on developed contexts where non-communicable diseases form the predominant burden of health. Mental health outcomes were included in 40 studies (one-third of all studies): 27 were at the neighbourhood level, and six and seven at the building and urban system scales, respectively. Only one-fifth of studies (27/124) reported some planetary health outcomes, and again mostly at the neighbourhood level (16/27), and 6/27 at the building and 5/27 at the urban system levels.

### 4.1 Where Next for Research?

This umbrella review indicates three potential directions for further research, more generally, and systematic review research, more specifically. First, research on urban health usually involves two distinct communities of scholars, health scientists and urban scientists, who can come from completely different research paradigms. This requires time to learn or synthetise across disciplines, transdisciplinary methods to account for the variety of entry points, but also relational thinking to acknowledge the multiple connections between the different elements of the urban system and the continuum of health outcomes, *e*.*g*. physical–mental–wellbeing. The studies identified by this paper come from teams of primarily health scientists, hence grounding findings with urban scientists can be challenging. There is also a predominant focus on the ‘negative psychology’ approach to health whereby treating the effects of a particular condition (*i.e*. cardiovascular, respiratory, BMI, blood pressure) is in focus, as opposed to ‘positive psychology’ approach when the cause of the condition (poverty/deprivation, vulnerability) is analysed (Seligman 2004). While some mental health and wellbeing outcomes are mentioned at the neighbourhood and wider urban system levels, they need better understanding. Here, a ‘flourish’ approach to health can be explored, whereby the focus is on people, rather than their health, under the assumption that improving the wider quality of life and social health would make for healthier people (Seligman 2011).

Second, there are three obvious gaps in the literature: the intersectionality of health outcomes, lack of evidence from low-income settings and little current discussion of communicable disease burden. While there are often clear pathways for increased risk for vulnerable population groups, review evidence for these groups appears to be limited and tends to be focused on children and the elderly; there is little consideration of how, *e*.*g*., age, gender and socio-economic status may intersect in the built environment and affect health. There was also very limited evidence from low-income countries and/or communicable disease outcomes; where reviews were identified they reported health outcomes associated with indoor air pollution from cooking stoves and informal living, lack of water infrastructure and sanitation, and vector-borne disease. The current COVID-19 pandemic will certainly move the focus back onto communicable disease outcomes.

Third, planetary health impacts are mainly reported in relation to carbon emissions. Expanding understanding beyond this point is another research direction worth exploring. Research reporting on planetary impacts in conjunction with human health ones, across all scales of the built environment, can reinforce advocacy for urban sustainability transitions. If the challenges of the health–climate crisis is to be met, the nexus of human and planetary health needs better understanding of unintended consequences, better policymaking and urban governance at all levels (Crane *et al*. 2021).

### 4.2 A Role for Policy and Urban Governance

Urban health research is closely associated with relevance to policymaking (Hawkes *et al*. 2016; Sallis *et al*. 2016; Schneidera & Blythb 2017). This paper found that many studies note implications for policymaking and urban stakeholders (*e*.*g*. urban planners, landscape architects, communities, residents, *etc*.), but discussion is rather general. This may be explained by the fact that most reviews take a health perspective whereby roles outside health in implementing or changing exposure risks in the built environment are not considered. In what follows, this paper contributes to expanding policy understanding in this area by exploring different types of policy interventions and actors involved across the scales of the built environment.

At the *building* level, exposure to many identified environmental risk factors is long-term and difficult to modify without substantial investment of time and resources. For example, remedial factors to address risks from exposure to low indoor temperatures via energy retrofitting to protect against winter cold may take substantial investment. Likewise, factors related to a building’s condition have clear benefits in terms of health when considering safety measure (*e*.*g*. injury from fire, falls, *etc.*) and building regulations usually address these issues, but regulation and compliance may be challenging in some settings or differ amongst sectors and professions. The building level is usually addressed by architects, designers, developers, building contractors, owners of individual structures and health practitioners.

At the *neighbourhood* level, the weight of evidence suggests positive health outcomes are associated with green space, although these may be confounded by socio-economic status, *i.e*. wealthier neighbourhoods having higher density of green space and living in deprived/poor neighbourhoods is linked to adverse health impacts for vulnerable groups such as children, the elderly and disable people, women and ethnic minority groups. While it may be challenging to add new green space to established cities, modifications to existing green space can be made to encourage physical activity, along with fair and equal access for all socio-economic groups and education to effect behaviour change. Actors involved in the governance of the neighbourhood level primarily include local government, local planning and local health trusts, communities, civil society and business organisations.

At the *urban system* level, the evidence presented points to at least two important ‘alignments’, *e*.*g*. between health, spatial planning and housing policy; and between climate change and health outcomes. City-level spatial planning policies can impact on health by providing adequate levels of affordable or social housing and so de-risking housing security, an important socio-economic determinant of health, while housing policy at the regional or national level can help to absorb shock-related health impacts from events such as the financial crisis in 2007–08 or the current COVID-19 pandemic. Furthermore, increasing greenspace and energy retrofitting are associated with clear health and climate change-positive outcomes; high density or shared living can reduce pressure on resources and associated carbon emissions, but some evidence suggests mental health-negative outcomes from overcrowding and impacts on physical health from limited space. The latter is easier to address in policy terms, *e*.*g*. the provision of easily accessible parks and recreational facilities to allow physical activity, while overcrowding is harder to address and requires long-term action and investment, but policy measures such as standards and regulations can help. The governance of the urban system involves all the actors at the building and neighbourhood level and much more, *i.e*. regional and national governments involved in strategic policymaking, but also global organisations including international institutions such as the WHO and European Union.

The above examples frame urban health within the wider process of policymaking in the build environment. Policymaking is a complex and, most importantly, a political process; it is not something happening at a particular time, in a particular spot, but part of wider multilevel governance frameworks, which frame the complexity of the urban system and local built environment. As seen above, the governance of urban health involves policymakers responsible for health- or housing-related policy and regulation, and other stakeholders involved in health interventions such as government agencies, architects, builders, housing providers, developers, engineers, urban planners, industry regulators, financial institutions as well as social services, community groups and public health professionals. These stakeholders are ultimately required to ensure that housing is built, maintained, renovated, used and demolished in ways that support health.

By taking a scale perspective on health, the connections between different policies and levels of policymaking become apparent. For example, national government needs to align health with SDGs, ensure geographical equity and combat siloed approaches, while local government must ensure that public health and spatial planning work together to strengthen the link between people and places and break administrative boundaries to reap the benefits of planetary health. Also, the wider the scale, the more complex the array of actors involved and the dynamic lines of power and networks inside and outside policymaking (Bulkeley & Kern 2006). Communities may hold important knowledge about public health in their locality, but may not have a voice, especially if marginalised; civil society organisations may lobby or support government and communities, while professional communities such as urban planners share many communalities with public health professionals including an interest in the public good, and use of evidencebased and long-term assessment approaches (WHO 2020). If evidence fails to engage with the multi-scalar, multilevel nature of urban governance, which makes links across scales from local to supranational, and where power is distributed across horizontal and vertical networks which do not operate in a hierarchical manner (Bulkeley & Betsill 2005). This paper suggests that a better grounding of urban health research in existing urban governance landscapes would not only support a faster and more efficient implementation of health interventions at the local level, but also gauge potential synergies and tensions at the urban system level with other pressing urban challenges such as climate change.

### 4.3 Strengths and Limitations

This paper is novel for synthesising evidence on housing from the perspective of scales within the urban built environment. Most research has either considered housing from a narrow understanding of the individual’s dwelling or disconnected the built environment from its purpose of providing liveability (Giles-Corti *et al*. 2019). To the present authors’ knowledge, this is the first umbrella review to take a comprehensive view of the local built environment and housing at scale while looking at both physical and socio-economic characteristics that define the complex urban system.

This review is limited to systematic review evidence and generic geographical regions. Evidence derived purposely for specific countries was excluded because of the specific content and context. As such, the authors acknowledge the broad conclusions made in this review. The intent was not to assess the size of health impacts or the effectiveness of interventions and make no assessment of the importance of one health determinant or built factor over another. Furthermore, the study excluded research on shelters for the homeless, residential care or student accommodation, and rural housing, which also pose built environment, societal and health concerns.

## 5 Conclusions

This paper provides an umbrella review of reported health impacts across three broad spatial scales and notes that most research has focused on health at the neighbourhood level. Discussions of planetary health and policy implications have been limited, and only a few studies have evaluated the economic implications of health interventions. This study is the first of its type: it applies a multiscalar perspective to health, it suggests directions for potential future research, and it expands the discussion of urban governance for health.

## Supplementary Material

Supplemental data for this article can be accessed at: https://doi.org/10.5334/bc.119.s1

Supplementary Table

Supplementary Table

Supplementary Table

## Figures and Tables

**Table 1:** Systematic reviews reporting on BUILDING determinants of health.

DETERMINANT	SPECIFIC ASPECTS	HUMAN HEALTH	PLANETARY HEALTH
Indoor environmental quality	*Air quality* (*e*.*g*. particles including radon, fungi, PM, NO_2_, VOC; and, chemicals)	Impacts of exposure on lung cancer (Ajrouche *et al*. 2017); cognitive and neurobehavioral outcomes (Nussbaumer-Streit *et al*. 2020); exacerbation of asthma (Sharpe *et al*. 2015; Tagiyeva & Sheikh 2014; Naldzhiev *et al*. 2020; Van Boven *et al*. 2020); respiratory and general health and wellbeing (Vardoulakis *et al*. 2020)	Air pollution (PM, NO_2_, VOCs, *etc.*) (Vardoulakis *et al*. 2020); energy demand (Naldzhiev *et al*. 2020)
	*Occupant hazards* (*e*.*g*. burning fuel, ventilation, room temperature)	Impacts of exposure on thermal control in the elderly and those with respiratory conditions (Jevons *et al*. 2016); cardio-respiratory, paediatric, maternal outcomes and mortality (Lee *et al*. 2020a; Katoto *et al*. 2019) Environmental equity (Bailey *et al*. 2019); air pollution (PM) (Katoto *et al*. 2019; Quansah *et al*. 2017); climate temperature (Jevons *et al*. 2016)
		Interventions to reduce exposure and general health benefits to children and adults (Quansah *et al*. 2017; Bailey *et al*. 2019)	
Dwelling condition	*Home improvements* (*e*.*g*. energy retrofit, upgrade of bathrooms and kitchens)	Injury and falls prevention, mobility, independence and wellbeing (Carnemolla & Bridge 2020); health economic benefits (Fenwick *et al*. 2013); reduction in depression in the elderly (Garin *et al*. 2014); respiratory outcomes, QoL and mental health (Ige *et al*. 2019); lung disease prevention (Pega & Wilson 2016)	Energy required for ventilation (Naldzhiev *et al*. 2020)
	*Soundness* (*e*.*g*. risk of fire, falls, structural integrity)	Interventions to reduce fire-related deaths and injuries avoided (Senthilkumaran *et al*. 2019; Pega & Wilson 2016)	
Dwelling design	*Green buildings* (*e*.*g*. green designs, green standards, green walls)	Impacts of interventions on: respiratory symptoms and general wellbeing (Allen *et al*. 2015); flood risk and outcomes such as waterborne diseases, mortality and psychological harm (Houghton & Castillo-Salgado 2019); and heat-related morbidity and mortality (Houghton & Castillo-Salgado 2017)	Reduced energy use and CO_2_ emissions (Allen *et al*. 2015); attention to the interface between humans, habitats, wildlife and water systems (Houghton & Castillo-Salgado 2017, 2019)
	*Building type* (*e*.*g*. high-rise, apartment, duplex, co-housing, accessible-by-design, size, shared sanitation)	Impacts of conditions on: social wellbeing, QoL and mental health (Barros *et al*. 2019; Carrere *et al*. 2020; Cho *et al*. 2016; Garin *et al*. 2014); sedentary lifestyles (Chastin *et al*. 2015); falls and mortality (Cho *et al*. 2016); infectious diseases and maternal outcomes (Heijnen *et al*. 2014)	Efficient use of resources (Barros *et al*. 2019)
	*Outdoor space* (*e*.*g*. gardens, outdoor equipment)	Interventions to improve the physical activity of children (Carlin *et al*. 2017); stress reduction (*e*.*g*. HR and BP) (Kondo *et al*. 2018b)	

*Note:* BP = blood pressure; CO_2_ = carbon dioxide; HR = heart rate; NO_2_ = nitrogen dioxide); PM = particulate matter; QoL = quality of life; VOC = volatile organic compounds.

**Table 2:** Systematic reviews reporting on NEIGHBOURHOOD determinants of health.

DETERMINANT	SPECIFIC ASPECTS	HUMAN HEALTH	PLANETARY HEALTH
Green and blue infrastructure	*Greenery* (*e*.*g*. green and natural space, contact with nature, green infrastructure, urban agriculture)	Impacts on: physical, mental and/or social health and mortality (Browning & Lee 2017; Calogiuri & Chroni 2014; Carmona 2019; Venkataramanan *et al*. 2019; van den Berg *et al*. 2015; de Keijzer *et al*. 2020; Dzhambov & Dimitrova 2018; Felappi *et al*. 2020; Shuvo *et al*. 2020; Gascon *et al*. 2015; Kondo *et al*. 2018a; Lai *et al*. 2019; Rojas-Rueda *et al*. 2019; Parker & de Baro 2019; Rugel & Brauer 2020; Macmillan *et al*. 2018; Audate *et al*. 2019), including for children (Vanaken & Danckaerts 2018; de Keijzer *et al*. 2016; Hartley *et al*. 2020; Islam *et al*. 2020; McCormick 2017), maternal health (Twohig-Bennett & Jones 2018; Lee *et al*. 2020c), elderly people (Yuan *et al*. 2020; Garin *et al*. 2014; Levy-Storms *et al*. 2018; Chastin *et al*. 2015), people with disabilities (Zhang *et al*. 2017; Saitta *et al*. 2019), and health inequalities (Schüle *et al*. 2019)	Ecosystem services, wildlife and biodiversity (Zhang *et al*. 2017; Felappi *et al*. 2020; Schüle *et al*. 2019; Lai *et al*. 2019; Parker & de Baro 2019); floods (Yuan *et al*. 2020); air pollution (Lee *et al*. 2020c); contributions to the SDGs (Vanaken & Danckaerts 2018)
	*Water collection and waste* (*e*.*g*. blue space, urban drains, sewage)	Health risks associated with floods (Ishaq *et al*. 2020) and diarrhoeal disease (Jung *et al*. 2017)	Floods (Ishaq *et al*. 2020)
Physical conditions	*Soundscape* (*e*.*g*. noise, including from traffic; noise buffers)	Hypertension (Van Kempen & Babisch 2012; Dzhambov & Dimitrova 2018), myocardial infarction (Khosravipour & Khanlari 2020), stress recovery and self-reported health (Aletta *et al*. 2018), disability in the elderly (Garin *et al*. 2014), as well as combined pathways to health (Peris & Fenech 2020)	Ecosystem responses to transport noise and natural environment impact on noise (Peris & Fenech 2020)
	*Urban design* (*e*.*g*. walkability, rest areas and benches, street layout and connectivity)	Physical health including hypertension, BMI and type 2 diabetes (Chandrabose *et al*. 2019; Leal & Chaix 2011; Malambo *et al*. 2016); physical activity in the elderly (Chastin *et al*. 2015; Moran *et al*. 2014) and people with disabilities (Eisenberg *et al*. 2017); child accidents (Schüle & Bolte 2015); mental health and QoL (Schüle & Bolte 2015; Garin *et al*. 2014)	Weather and environmental conditions (Moran *et al*. 2014)
	*Neighbourhood renewal* (*e*.*g*. improvement, upgrade, renewal)	Impacts on socio-economic determinants of health (Thomson & Thomas 2015), and mental health and wellbeing (Moore *et al*. 2018)	
Transport	*Traffic exposure* (*e*.*g*. measures, street design)	Morbidity and mortality associated with cardiovascular, respiratory, metabolic and reproductive health (Hamra *et al*. 2015; Rugel & Brauer 2020; Malambo *et al*. 2016), including child health for lung function (Barone-Adesi *et al*. 2015), leukaemia (Boothe *et al*. 2014) and obesity (Audrey & Batista-Ferrer 2015)	Air pollution (Barone-Adesi *et al*. 2015; Hamra *et al*. 2015; Rugel & Brauer 2020)
	*Travel mode* (*e*.*g*. public transportation, cycling, walking)	Changes in cycling behaviour (Stewart *et al*. 2015); health associated with physical activity, air pollution and injuries (Stankov *et al*. 2020; Patterson *et al*. 2019; Möller *et al*. 2020; Macmillan *et al*. 2018); health of children from disadvantaged socio-economic groups (Ma *et al*. 2020)	Air pollution (Möller *et al*. 2020) and environmental justice (Ma *et al*. 2020)
	*Mobility* (*e*.*g*. transport barriers and restrictions)	Premature mortality in the elderly (Rosso *et al*. 2011) and congenital heart disease (Davey *et al*. 2020)	
Access to local services	*Shops* (*e*.*g*. grocery, supermarkets, farmer markets, community kitchen, retail)	Physical activity- and dietary-related health outcomes including blood pressure, BMI, type 2 diabetes, mental health and self-reported health (Abeykoon *et al*. 2017; Garin *et al*. 2014; Macmillan *et al*. 2018; Malambo *et al*. 2016; Iacovou *et al*. 2013)	
	*Healthcare* (*e*.*g*. primary care)	Congenital heart disease (Davey *et al*. 2020)	
	*Education* (*e*.*g*. schools)	Health of children from disadvantaged socio-economic groups (Ma *et al*. 2020)	Environmental justice (Ma *et al*. 2020)
Socio-economic conditions	*Disadvantage* (*e*.*g*. deprivation, segregation, gentrification)	Impacts on self-reported health, mental health and health-related behaviours (Algren *et al*. 2015; Bécares *et al*. 2018; Bhavsar *et al*. 2020)	Environmental equity (Bhavsar *et al*. 2020)
	*Social capital* (*e*.*g*. time spent with others, leisure activities, social cohesion)	Physical activity, mental and social health (Lee *et al*. 2020b; Rhodes *et al*. 2018; Pérez *et al*. 2020), including benefits for children and adolescents (Vyncke *et al*. 2013)	
	*Risk* (*e*.*g*. unsafe environment, crime)	Earlier sexual initiation and increased adolescent pregnancy (Decker *et al*. 2018); mental health risks due to a fear of crime (Lorenc *et al*. 2013)	

*Note:* BMI = body mass index, QoL = quality of life, SDG = Sustainability Development Goals.

**Table 3:** Systematic reviews reporting on URBAN SYSTEM determinants of health.

DETERMINANT	SPECIFIC ASPECTS	HUMAN HEALTH	PLANETARY HEALTH
Spatial planning	*Informality* (*e*.*g*. slums)	Physical and mental health, as well as QoL and social capital (Alaazi & Aganah 2020; Turley *et al*. 2013; Henson *et al*. 2020)	Rapid urbanisation and effects on the environment and SDGs (Henson *et al*. 2020)
	*Urban infrastructure* (*e*.*g*. water, power lines, urban structure)	Morbidity and mortality, including gastroenteritis, cancer, CVD, reproductive outcomes, and neurogenerative disease, and risk of displacement following earthquakes (Beaudeau 2018; Doocy *et al*. 2013; Habash *et al*. 2019)	
	*Type of development* (*e*.*g*. densification, built-form typology, urbanicity)	Health-related behaviours, including physical activity and fruit and vegetable consumption, and associated outcomes such as obesity, cardiometabolic diseases and mental health (Berghauser Pont *et al*. 2020; Chandrabose *et al*. 2019)	Densification contributions to the SDGs (Berghauser Pont *et al*. 2020)
	*Master planning* (*e*.*g*. urban designing for: active ageing, healthcare access, smart growth, smart city, landscaping)	Physical activity (Nordbø *et al*. 2018; McCrorie *et al*. 2014; Durand *et al*. 2011; Rocha *et al*. 2019), mental health (Gong *et al*. 2016), including the health of children and the elderly (Sánchez-González *et al*. 2020; Kabisch *et al*. 2017), and morbidity and mortality (Salgado *et al*. 2020)	Air pollution, noise, UHI, green and blue space (Salgado *et al*. 2020; Kabisch *et al*. 2017; Rocha *et al*. 2019)
Housing system	*Vulnerability* (*e*.*g*. housing status, insecurity and instability; social housing)	Physical health, mental health and health-related behaviours (Vásquez-Vera *et al*. 2017; Downing 2016; Tsai 2015; Singh *et al*. 2019; Davey *et al*. 2020; Aidala *et al*. 2016), as well as health-related social outcomes (Aubry *et al*. 2020; Tanner *et al*. 2013)	Energy access and fuel poverty (Tanner *et al*. 2013)
	*Policy* (*e*.*g*. housing support, healthy municipality strategy)	Diabetes (Singh *et al*. 2019), obesity (Tseng *et al*. 2018), and community health status (Chaparro *et al*. 2020)	
Ecosystems	*Air* (*e*.*g*. pollution)	Mortality, cardiorespiratory health and health equity (Benmarhnia *et al*. 2014; Katoto *et al*. 2019)	Air pollution (Katoto *et al*. 2019; Gascon *et al*. 2017)
	*Water* (*e*.*g*. exposure to blue space)	Mental health (Gascon *et al*. 2017)	
	*Climate* (*e*.*g*. microclimate, UHI)	All-cause mortality and cardiorespiratory morbidity (Schinasi *et al*. 2018)	Climate change (Schinasi *et al*. 2018)

*Note:* CVD = cardiovascular disease; QoL = quality of life; SDGs = Sustainability Development Goals; UHI = urban heat island.

## Policy Relevance

Evidence about local built environment determinants of health have focused to date on physical health outcomes and the neighbourhood level; there is also significant evidence about mental health outcomes and greenspace. Future research can strengthen understanding of mental health outcomes across all scales and determinants of the built environment; investigate more robustly planetary health outcomes; and provide additional evidence at the building and wider urban system level, especially in relation to low-income settings, vulnerable groups and communicable disease. There is limited discussion of implications for policymaking and economic evaluations of health interventions, *i.e*. the cost of intervention *versus* health outcomes. Urban health interventions have focused to date on treating the *effects* of health conditions; however, there is potential for constructive interventions in the built environment (at various scales) which improve health and/or reduce environmental health risks if the policy focus is on dealing with the *causes* of health conditions.
