# Supplementary material for: A multi-scalar perspective on health and urban housing: an umbrella review
Source: Build Cities. Author manuscript; Available in PMC 2021 Nov 3. (PMC7611930; doi:10.5334/bc.119)
Supplement: Supplementary Table [file EMS137021-supplement-Table_S1.docx]

Table 1 – Systematic reviews reporting on DWELLING determinants of health

| *Determinant* | *Specific aspects* | *Human health impact or outcomes reported* | *Planetary health impacts or outcomes* | *Reference* |
| --- | --- | --- | --- | --- |
| **Indoor environmental quality** | **Air quality**  (e.g. radon, fungi, pollutant particles, VOC, SVOC, lead etc) | Indoor radon exposure AND lung cancer incidence. | - | (Ajrouche et al. 2017) |
|  |  | Exposure to indoor fungi (Penicillium, Aspergillus, Cladosporium and Alternaria species) AND asthma outcomes in children and adults. | - | (Sharpe et al. 2015) |
|  |  | Indoor air pollution from proximity to high-traffic-density roads, household products, and small house size AND respiratory and general health and wellbeing outcomes | air pollution (PM, NO2, VOCs etc) | (Vardoulakis et al. 2020) |
|  |  | Use of household air purification interventions to reduce asthma exacerbation | - | (Van Boven et al. 2020) |
|  |  | Domestic VOC exposure AND asthma and allergy outcomes in children and adults | - | (Tagiyeva and Sheikh 2014) |
|  |  | Lead exposure and effects on cognitive and neurobehavioral outcomes | - | (Nussbaumer-Streit et al. 2020) |
|  |  | Exposure to VOCs and SVOC and other chemicals from home insulation products such as foam sprays AND impacts on health are not conclusive. | energy required for ventilation | (Naldzhiev, Mumovic, and Strlic 2020) |
|  | **Occupant hazards**  (e.g. burning, cooking, heating etc) | Reducing wood burning emissions AND human health co-benefits (health disparities) | improving environmental equity | (Bailey et al. 2019) |
|  |  | Biomass burning in homes AND cardio-respiratory outcomes in children, adults and older adults. | air pollution (PM) | (Katoto et al. 2019) |
|  |  | Household air pollution from cooking or heating AND cardio-respiratory, paediatric, and maternal outcomes and no of deaths. | - | (Lee et al. 2020) |
|  |  | Cooking stove interventions on household air pollution AND outcomes in children and adults. | air pollution (PM) | (Quansah et al. 2017) |
|  |  | Cold homes AND blood pressure, reduced thermal control in the elderly and those with respiratory conditions. | climate temperature | (Jevons et al. 2016) |
| **Dwelling condition** | **Home improvements** (e.g. layout reconfiguration, energy efficiency, lead removal,insulation etc) | Home improvements AND injury and falls prevention; improved function, self-care or independence; physical health and wellbeing. | - | (Carnemolla and Bridge 2020) |
|  |  | Home improvement AND economic analysis of the health impacts (i.e. general health; mental health; respiratory health; and other/illness and symptom). | - | (Fenwick, Macdonald, and Thomson 2013) |
|  |  | satisfaction with dwelling quality AND depression in the elderly | - | (Garin et al. 2014) |
|  |  | Housing refurbishment and modifications, provision of adequate heating, improvements to ventilation and water supply AND improved respiratory outcomes, QoL and mental health. | - | (Ige et al. 2019) |
|  |  | Cost benefit of removing indoor lead and retrofitting insulation AND lung disease | - | (Pega and Wilson 2016) |
|  |  | Emissions from spray foam products used in home insulation AND health impacts | Energy required for ventilation | (Naldzhiev, Mumovic, and Strlic 2020) |
|  | **Soundness** (e.g. structure, fire etc) | Home fire safety interventions AND fire-related deaths avoided | - | (Senthilkumaran et al. 2019) |
|  |  | Improving home safety and providing smoke alarms AND home injuries | - | (Pega and Wilson 2016) |
| **Dwelling design** | **Green buildings**  **(e.g. green walls, green design, green standards etc)** | Green building design AND self-reported wellbeing and respiratory symptoms. | reduce energy use and CO2 emissions | (Allen et al. 2015) |
|  |  | Green buildings AND the risk of waterborne disease, flood-related morbidity, mortality, and psychological harm. | the interface between humans and wildlife, flooding | (Houghton and Castillo-Salgado 2019) |
|  |  | Aspects of green buildings (site selections; density and connectivity; habitat restoration/protection; open space; storm water design; heat island via roof/nonroof; onsite renewable energy; indoor AQ) AND health-related outcomes (i.e. heat-related morbidity, mortality; risk of cardio-respiratory, malnutrition, and mental health). | co-benefits: habitat restoration, water renewable energy | (Houghton and Castillo-Salgado 2017) |
|  | **Building type**  (e.g. high-rise, apartment, duplex, cohousing, accessible-by-design, size, shared sanitation etc) | House type, floor level, as well as spaces intrinsic to high-rise residential buildings (e.g. shared stairwells) AND social well-being and mental health | efficient use of resources | (Barros et al. 2019) |
|  |  | Apartment or duplex living AND sedentary lifestyles. | - | (Chastin et al. 2015) |
|  |  | Cohousing AND physical and mental health, QoL and wellbeing | - | (Carrere et al. 2020) |
|  |  | Accessible-by-design homes (for people with functional limitations/ impairment) AND falls, mortality rates and mental health |  | (Cho et al. 2016) |
|  |  | Housing type, size, rooms AND depression, QoL, wellbeing, life satisfaction and injury in aging. | - | (Garin et al. 2014) |
|  |  | Sharing sanitation/ latrines results AND diarrhoea, helminth infections, enteric fevers, other faecal-oral diseases, trachoma and adverse maternal or birth outcomes. | - | (Heijnen et al. 2014) |
|  | **Outdoor space**  (e.g. gardens, outdoor equipment etc) | Backyard garden/ outdoor equipment AND PA associated health benefits in children. | - | (Carlin et al. 2017) |
|  |  | Time spent gardening AND stress (HR, BP) | - | (Kondo, Jacoby, and South 2018) |

Legend: BP (Blood pressure) BMI (body mass index), CO2 (Carbon dioxide), CVD (Cardiovascular Disease), ED (Emergency department), F&V(fruit and vegetable), HR (heart rate), NO2 (Nitrogen Dioxide), PA (physical activity), PM (particulate matter), QoL (quality of life), SDG (Sustainability Development Goals), Urban Heat Islands (UHI), VOC (Volatile Organic Compounds)

Ajrouche, R., G. Ielsch, E. Cléro, C. Roudier, D. Gay, J. Guillevic, D. Laurier, and A. Le Tertre. 2017. 'Quantitative Health Risk Assessment of Indoor Radon: A Systematic Review', *Radiation protection dosimetry*, 177: 69-77.

Allen, J. G., P. MacNaughton, J. G. C. Laurent, S. S. Flanigan, E. S. Eitland, and J. D. Spengler. 2015. 'Green Buildings and Health', *Current environmental health reports*, 2: 250-58.

Bailey, J., E. Gerasopoulos, D. Rojas-Rueda, and T. Benmarhnia. 2019. 'Potential health and equity co-benefits related to the mitigation policies reducing air pollution from residential wood burning in Athens, Greece', *Journal of Environmental Science and Health - Part A Toxic/Hazardous Substances and Environmental Engineering*, 54: 1144-51.

Barros, P., L. Ng Fat, L. M. T. Garcia, A. D. Slovic, N. Thomopoulos, T. H. de Sá, P. Morais, and J. S. Mindell. 2019. 'Social consequences and mental health outcomes of living in high-rise residential buildings and the influence of planning, urban design and architectural decisions: A systematic review', *Cities*, 93: 263-72.

Carlin, A., C. Perchoux, A. Puggina, K. Aleksovska, C. Buck, C. Burns, G. Cardon, S. Chantal, D. Ciarapica, G. Condello, T. Coppinger, C. Cortis, S. D’Haese, M. De Craemer, A. Di Blasio, S. Hansen, L. Iacoviello, J. Issartel, P. Izzicupo, L. Jaeschke, M. Kanning, A. Kennedy, J. Lakerveld, F. C. M. Ling, A. Luzak, G. Napolitano, J. A. Nazare, T. Pischon, A. Polito, A. Sannella, H. Schulz, R. Sohun, A. Steinbrecher, W. Schlicht, W. Ricciardi, C. Macdonncha, L. Capranica, and S. Boccia. 2017. 'A life course examination of the physical environmental determinants of physical activity behaviour: A “Determinants of Diet and Physical Activity” (DEDIPAC) umbrella systematic literature review', *PLoS ONE*, 12.

Carnemolla, P., and C. Bridge. 2020. 'A scoping review of home modification interventions – Mapping the evidence base', *Indoor and Built Environment*, 29: 299-310.

Carrere, J., A. Reyes, L. Oliveras, A. Fernández, A. Peralta, A. M. Novoa, K. Pérez, and C. Borrell. 2020. 'The effects of cohousing model on people's health and wellbeing: A scoping review', *Public Health Reviews*, 41.

Chastin, S. F. M., C. Buck, E. Freiberger, M. Murphy, J. Brug, G. Cardon, G. O'Donoghue, I. Pigeot, J. M. Oppert, and Dedipac consortium on behalf of the. 2015. 'Systematic literature review of determinants of sedentary behaviour in older adults: A DEDIPAC study', *International Journal of Behavioral Nutrition and Physical Activity*, 12.

Cho, H. Y., M. MacLachlan, M. Clarke, and H. Mannan. 2016. 'Accessible home environments for people with functional limitations: A systematic review', *International Journal of Environmental Research and Public Health*, 13.

Fenwick, E., C. Macdonald, and H. Thomson. 2013. 'Economic analysis of the health impacts of housing improvement studies: A systematic review', *Journal of Epidemiology and Community Health*, 67: 835-45.

Garin, N., B. Olaya, M. Miret, J. L. Ayuso-Mateos, M. Power, P. Bucciarelli, and J. M. Haro. 2014. 'Built environment and elderly population health: A comprehensive literature review', *Clinical Practice and Epidemiology in Mental Health*, 10: 103-15.

Heijnen, M., O. Cumming, R. Peletz, G. K. S. Chan, J. Brown, K. Baker, and T. Clasen. 2014. 'Shared sanitation versus individual household latrines: A systematic review of health outcomes', *PLoS ONE*, 9.

Houghton, A., and C. Castillo-Salgado. 2017. 'Health co-benefits of green building design strategies and community resilience to urban flooding: A systematic review of the evidence', *International Journal of Environmental Research and Public Health*, 14.

———. 2019. 'Associations between green building design strategies and community health resilience to extreme heat events: A systematic review of the evidence', *International Journal of Environmental Research and Public Health*, 16.

Ige, J., P. Pilkington, J. Orme, B. Williams, E. Prestwood, D. Black, L. Carmichael, and G. Scally. 2019. 'The relationship between buildings and health: A systematic review', *Journal of Public Health (United Kingdom)*, 41: E121-E32.

Jevons, R., C. Carmichael, A. Crossley, and A. Bone. 2016. 'Minimum indoor temperature threshold recommendations for English homes in winter – A systematic review', *Public Health*, 136: 4-12.

Katoto, P. D. M. C., L. Byamungu, A. S. Brand, J. Mokaya, H. Strijdom, N. Goswami, P. De Boever, T. S. Nawrot, and B. Nemery. 2019. 'Ambient air pollution and health in Sub-Saharan Africa: Current evidence, perspectives and a call to action', *Environmental Research*, 173: 174-88.

Kondo, M. C., S. F. Jacoby, and E. C. South. 2018. 'Does spending time outdoors reduce stress? A review of real-time stress response to outdoor environments', *Health and Place*, 51: 136-50.

Lee, K. K., R. Bing, J. Kiang, S. Bashir, N. Spath, D. Stelzle, K. Mortimer, A. Bularga, D. Doudesis, S. S. Joshi, F. Strachan, S. Gumy, H. Adair-Rohani, E. F. Attia, M. H. Chung, M. R. Miller, D. E. Newby, N. L. Mills, D. A. McAllister, and A. S. V. Shah. 2020. 'Adverse health effects associated with household air pollution: a systematic review, meta-analysis, and burden estimation study', *The Lancet Global Health*, 8: e1427-e34.

Naldzhiev, D., D. Mumovic, and M. Strlic. 2020. 'Polyurethane insulation and household products – A systematic review of their impact on indoor environmental quality', *Building and Environment*, 169.

Nussbaumer-Streit, B., V. Mayr, A. I. Dobrescu, G. Wagner, A. Chapman, L. M. Pfadenhauer, S. Lohner, S. K. Lhachimi, L. K. Busert, and G. Gartlehner. 2020. 'Household interventions for secondary prevention of domestic lead exposure in children', *Cochrane Database of Systematic Reviews*, 2020.

Pega, F., and N. Wilson. 2016. 'A systematic review of health economic analyses of housing improvement interventions and insecticide-treated bednets in the home', *PLoS ONE*, 11.

Quansah, R., S. Semple, C. A. Ochieng, S. Juvekar, F. A. Armah, I. Luginaah, and J. Emina. 2017. 'Effectiveness of interventions to reduce household air pollution and/or improve health in homes using solid fuel in low-and-middle income countries: A systematic review and meta-analysis', *Environment International*, 103: 73-90.

Senthilkumaran, M., G. Nazari, J. C. MacDermid, K. Roche, and K. Sopko. 2019. 'Effectiveness of home fire safety interventions. A systematic review and metaanalysis', *PLoS ONE*, 14.

Sharpe, R. A., N. Bearman, C. R. Thornton, K. Husk, and N. J. Osborne. 2015. 'Indoor fungal diversity and asthma: A meta-analysis and systematic review of risk factors', *Journal of Allergy and Clinical Immunology*, 135: 110-22.

Tagiyeva, N., and A. Sheikh. 2014. 'Domestic exposure to volatile organic compounds in relation to asthma and allergy in children and adults', *Expert Review of Clinical Immunology*, 10: 1611-39.

Van Boven, F. E., N. W. De Jong, G. J. Braunstahl, L. R. Arends, and R. Gerth Van Wijk. 2020. 'Effectiveness of the Air Purification Strategies for the Treatment of Allergic Asthma: A Meta-Analysis', *International Archives of Allergy and Immunology*, 181: 395-402.

Vardoulakis, S., E. Giagloglou, S. Steinle, A. Davis, A. Sleeuwenhoek, K. S. Galea, K. Dixon, and J. O. Crawford. 2020. 'Indoor exposure to selected air pollutants in the home environment: A systematic review', *International Journal of Environmental Research and Public Health*, 17: 1-24.
