# Supplementary material for: A multi-scalar perspective on health and urban housing: an umbrella review
Source: Build Cities. Author manuscript; Available in PMC 2021 Nov 3. (PMC7611930; doi:10.5334/bc.119)
Supplement: Supplementary Table [file EMS137021-supplement-Table_S2.docx]

**Health and urban housing: a multi-scalar perspective**

**Catalina Turcu** (corresponding author)

The Bartlett School of Planning

Faculty of the Built Environment

University College London, U.K.

[Catalina.turcu@ucl.ac.uk](mailto:Catalina.turcu@ucl.ac.uk)

ORCID 0000-0003-2663-2586

**Melanie Crane**

The University of Sydney

Sydney School of Public Health

The Charles Perkins Centre

Sydney, Australia

[Melanie.crane@sydney.edu.au](mailto:Melanie.crane@sydney.edu.au)

ORCID 0000-0002-3058-2211

**Emma Hutchinson**

Public Health, Environments and Society

Faculty of Public Health and Policy

London School of Hygiene and Tropical Medicine, U.K.

[Emma.Hutchinson@lshtm.ac.uk](mailto:Emma.Hutchinson@lshtm.ac.uk)

ORCID 0000-0003-0618-0068

**Simon Lloyd**

Climate and Health Programme (CLIMA)

Barcelona Institute for Global Health (ISGlobal)

Barcelona, Spain

[simon.lloyd@isglobal.org](mailto:simon.lloyd@isglobal.org)

ORCID 0000-0002-9728-8674

**Kristine Belasova**

Public Health, Environments and Society

Faculty of Public Health and Policy

London School of Hygiene & Tropical Medicine, U.K.

[Kristine.Belesova@lshtm.ac.uk](mailto:Kristine.Belesova@lshtm.ac.uk)

ORCID 0000-0002-6160-5041

**Paul Wilkinson**

Public Health, Environments and Society

Faculty of Public Health and Policy

London School of Hygiene &Tropical Medicine, UK

[paul.wilkinson@lshtm.ac.uk](mailto:paul.wilkinson@lshtm.ac.uk)

ORCID 0000-0001-7456-259X

**Mike Davies**

UCL Institute for Environmental Design and Engineering

Faculty of the Built Environment

University College London, U.K.

[Michael.davies@ucl.ac.uk](mailto:Michael.davies@ucl.ac.uk)

ORCID 0000-0003-2173-7063

# Abstract

With more than half of the world’s population living in cities, understanding how the built environment impacts human health at different urban scales is crucial. To be able to shape cities for health, we also need to understand the planetary health impacts, which encompass the human health impacts of human-caused disruptions on Earth's natural ecosystems. This umbrella review maps health evidence across the spatial scales of the built environment (building; neighbourhood; and wider system including city, regional and planetary levels), with a specific focus on urban housing. Systematic reviews published in English between January 2011 to December 2020 were searched across 20 databases, with 1176 articles identified and 124 articles screened for inclusion. Findings suggests that most evidence reports on health determinants at the neighbourhood level such as greenspace, physical and socio-economic conditions, transport infrastructure and access to local services. Physical health outcomes are also primarily reported, with an emerging interest in mental health outcomes. There is little evidence on planetary health outcomes.

# Policy relevance

Evidence about local built environment determinants of health have focused to date on physical health outcomes and the neighbourhood level; there is also significant evidence about mental health outcomes and greenspace. Future research should strengthen understanding of mental health outcomes across all scales and determinants of the built environment; investigate more robustly planetary health outcomes; and provide additional evidence at the building and wider urban system level, especially in relation to low-income settings, vulnerable groups and communicable disease. There is limited discussion of implications for policymaking and economic evaluations of health interventions i.e. cost of intervention vs health outcomes. Urban health interventions have focused to date on treating the *effects* of health conditions; however, there is potential for constructive interventions in the built environment (at various scales) which improve health and/or reduce environmental health risks if the policy focus is on dealing with the *causes* of health conditions.

# Key words

Built environment; Health; Urban; Housing; Building; Neighbourhood

# Introduction

The influence of the urban built environment on human health is complex and multifaceted. Health is determined by local environmental exposure and behaviours of individuals, which are socially- and spatially-patterned ([Marmot 2005](#_ENREF_85)). Material aspects of the built environment gives rise to various social processes that may directly and indirectly shape the health of its residents ([Rydin et al. 2012](#_ENREF_107); [Bai et al. 2016](#_ENREF_13)) . This poses difficulties to both conceptualising the relation between health and the urban built environment, and when seeking to design and implement actions to protect and improve health.

The urban built environment may be broadly defined at three scales: building, neighbourhood, and the wider urban system encompassing processes that operate across the city- and regional-levels and extend as far as the planetary-level. Addressing health at each spatial scale involves different types of planning and stakeholders, as well as governance frameworks. Hence, understanding the confluence of different scales is necessary to improve knowledge and actions in local urban environments ([Ramaswami et al. 2016](#_ENREF_101)). A multi-scalar perspective of the local built environment provides a comprehensive view of how health interventions may be implemented more efficiently at a particular scale. Multiple health objectives and outcomes, as well as health risks, may be considered simultaneously across the spectrum of policymaking and systematically reflected in decision-making. This requires a thorough understanding of the inter-relations between the different scales of the built environment in which a policy intervenes and this reaches beyond the local level and across social and political boundaries. Ultimately, local-level health considerations should be linked to the health of the wider urban system, including planetary health whereby environmental processes can both be disturbed by and threaten to disturb local living conditions and health ([Rydin et al. 2012](#_ENREF_107); [Ramaswami et al. 2016](#_ENREF_101); [Whitmee et al. 2015](#_ENREF_142)).

Urban housing is a primary area for health intervention in the built environment. Current urbanisation estimates suggest 68% of world’s population will live in cities by 2050. At the urban level, housing covers on average 70% of land-use thus, it is a substantial sector for policymaking ([UN 2018](#_ENREF_133)). Traditionally, approaches to research supporting health-focused policy action in the housing sector have considered dwellings as atomised units of exposure ([Thomson and Thomas 2015](#_ENREF_127); [Ige et al. 2019](#_ENREF_65)), although some recent work has begun to consider wider connections ([Bird et al. 2018](#_ENREF_23); [Carlin et al. 2017](#_ENREF_29); [Núñez-González et al. 2020](#_ENREF_93); [Pérez et al. 2020](#_ENREF_98)). This paper aims to synthese the current evidence on the built environment and health nexus, focusing on the urban housing sector as the entry-point. The paper adopts the World Health Organisation (2018) relational definition of housing and includes the physical structure of the dwelling; its immediate environment; and the community ([WHO 2018](#_ENREF_143)). Many systematic reviews already exist of the health impact of interventions and/or risks within the house or built environment. However, addressing the broader issues for policy implementation and decision-making across scales requires a broad synthesis of the evidence. Umbrella reviews are useful for efficiently summarising and comparing the evidence, assessing such considerations of aspects of health across built scales and identifying research gaps ([Aromataris and Munn 2020](#_ENREF_9)).

# Methods

## 2.1 Literature Search strategy

The Umbrella Review search strategy involved identifying peer-reviewed systematic reviews, published in the English language only, January 2011 to December 2020. To ensure that both health and urban studies research is captured, 20 databases were included: MEDLINE, Academic Search Ultimate, CINAHL, Health Source Nursing/academic Edition, Family and Society Studies Worldwide, Environment Complete, Sociology Source Ultimate, GreenFILE, Engineering Source, Psychology and Behavioral Sciences Collection, Rehabilitation and Sports Medicine, Business Source Ultimate, Applied Science and Technology, Health Business elite, MasterFILE complete, Women’s studies international, Legal source, LGBTQ+ source and British Education Index, as well as Scopus.

The search strategy was based on the intersection (Boolean AND) of terms for housing and its area of influence at the local and built scale (built environment OR slum* OR favella OR dwelling OR neighbourhood* OR house OR housing OR urban OR green space OR metropol* OR residen* OR accommodation OR informal settlement*))) OR (AB ((housing OR city OR cities OR urban OR municipal OR environment*) N4 (plan* OR strateg* OR design* OR infrastructure) and terms for health (health N4 (outcome* OR effect* OR assessment* OR benefit* OR gain* OR cost* OR impact* OR hazard*)). Health terms were broad in order to identify a wide spectrum of health outcomes including those relating to planetary health (including health effects of environmental pollution, climate change, water scarcity).

## 2.2 Eligibility criteria

Housing as defined here ([WHO 2018](#_ENREF_143)) included the dwelling and its immediate and wider surroundings in addition, such as the neighbourhood services and facilities necessary for residential living including green space, transportation, access to public services and facilities, and their connections to the city. Specific categories of housing such as student or elderly accommodation, residential care and shelters for the homeless were excluded. Systematic review articles published between January 2011 and December 2020 were eligible. Articles included meet the Cochrane Reviews criteria for systematic reviews: (1) clearly stated set of objectives with predefined eligibility criteria for included studies; (2) an explicit methodology; (3) a systematic search used to identify included studies; (4) an assessment of the validity of findings; and (5) a systematic presentation of results ([Higgins et al. 2019](#_ENREF_61)).

## 2.3 Data extraction

From the initial number of 2413 articles found, 1029 duplicates and 208 studies published prior to January 2011 were removed, leaving 1176 for manual screening of titles and abstracts by authors (Figure 1). Articles were rated by two reviewers independently. 264 articles were selected for full text review, of which 124 were agreed by the reviewers to meet the inclusion criteria and thus included in this umbrella review. The reasons for exclusion are summarized in the PRISMA diagram below (Figure 1).

[Figure 1 – insert here]

## 2.4 Quality assessment

The quality of included studies was assessed using the JBI manual for evidence synthesis of umbrella reviews ([Aromataris and Munn 2020](#_ENREF_9)). The following aspects were recorded: the topic or intervention of each paper, the context or geographical location, reported outcomes, study type (i.e. experimental or observational studies) and a critical assessment of how the review evidence was synthesised. Information was also collated on 1) the built scale at which the study was focused (building, neighbourhood, urban system); 2) any mentioned of planetary outcomes or links to planetary outcomes; and 3) stakeholders (i.e. public/private stakeholders at a local, city, regional or national level) and governance mechanisms (i.e. policy, regulation, legislation, standard or guidance).

# Results

## 3.1 Description of study quality and characteristics

The 124 articles primarily included research from high income countries, (predominantly the UK, Europe and North America); few included studies from low-income countries ([Alaazi and Aganah 2020](#_ENREF_5); [Henson et al. 2020](#_ENREF_60); [Jung et al. 2017](#_ENREF_69); [Katoto et al. 2019](#_ENREF_71); [Quansah et al. 2017](#_ENREF_100); [Shuvo et al. 2020](#_ENREF_120); [Turley et al. 2013](#_ENREF_131)) and these were mainly in relation to urban slum interventions or impacts. The types of studies included in each review were diverse, but mainly collated evidence from cross-sectional population surveys. The majority of reviews assessed the quality of evidence in terms of potential publication bias and used criteria for appraising studies for inclusion. While the majority commented on the poor quality and potential bias of included papers, quality was not always stated. Few reported research from randomized controlled trials or quasi-experimental studies and yet the quality of studies was generally reported by authors as medium quality. Study designs were generally broad, inclusive of qualitative and non-experimental evidence. The built environment was often broadly defined although a few studies incorporated GIS indicators when assessing outcomes at the neighbourhood or city scale ([Gong et al. 2016](#_ENREF_54); [Ma et al. 2020](#_ENREF_82); [Malambo et al. 2016](#_ENREF_84); [McCrorie, Fenton, and Ellaway 2014](#_ENREF_87); [Nordbø et al. 2018](#_ENREF_92)).

The majority of studies focused on the neighbourhood scale (n=80), followed by the dwelling (31) and urban system (n=32) level. Some studies addressed multiple scales but few identified these from a scale perspective ([Levy-Storms, Chen, and Loukaitou-Sideris 2018](#_ENREF_80)). Most focused on non-communicable disease and physical health, especially at the neighbourhood level; mental health was mostly examined at the neighbourhood level. At the dwelling level, physical and mental health outcomes featured with a greater focus on cardio-respiratory outcomes related to indoor air pollution and other hazards. Many studies focused on the general population but some were of more specific populations, including children (n= 20), older adults (n= 9), mothers and pregnant women (n= 3), the socially disadvantaged (n=5) or people with a specific chronic disease (i.e. HIV, diabetes, asthma) (n=4). A narrative synthesis of the findings follows below, structured under the three broad levels of housing at scale: building, neighbourhood and urban system. Figure 2-4 show the number of studies identified at each spatial level.

[Figure 2-4 – insert here]

## 3.2 Building determinants of health (31 reviews)

Thirty one (25%) of the systematic reviews reported factors that affect health at the building or dwelling level under three broad areas: ***indoor environmental quality*** (12 studies) such as *air quality* (e.g. indoor pollution from location and construction materials) and *occupant behaviour (*e.g. burning fuel, cooking, heating); ***dwelling condition*** (8 studies) such as *home improvements* (e.g. energy retrofits, bathroom/kitchens) and *soundness* (e.g. collapse and fire hazard); and ***dwelling design*** (11 studies) including *green buildings* (e.g. green walls, green design and green standards), *building type* (e.g. high-rise, apartment, cohousing, shared facilities) and *outdoor space* (e.g. gardens) (see Table 1).

[Table 1 – insert here; see end of this document and Supplementary Table 1]

3.2.1 Indoor environmental quality

Twelve systematic reviews were included in this category looking at *air quality* (7 studies) and *occupant hazards* (5 studies). The *air quality* sub-category included studies of outdoor air pollution in the vicinity of the dwelling (e.g. originating from such sources as a nearby traffic) ([Vardoulakis et al. 2020](#_ENREF_138)), its infiltration into the dwelling, and the associated risks for the cardio-vascular and respiratory health of the residents, including asthmatic and allergic exacerbations ([Tagiyeva and Sheikh 2014](#_ENREF_125); [Van Boven et al. 2020](#_ENREF_134)). The extent to which outdoor air pollution enters the dwelling is related to the building structure, its fabric, operation and ventilation/air purification technologies ([Van Boven et al. 2020](#_ENREF_134)). This sub-category also included studies of radon, lead, volatile and semi-volatile organic compounds (VOCs, SVOCs) ([Ajrouche et al. 2017](#_ENREF_3); [Naldzhiev, Mumovic, and Strlic 2020](#_ENREF_91); [Nussbaumer-Streit et al. 2020](#_ENREF_94)) as well as of biological agents ([Sharpe et al. 2015](#_ENREF_119)).The *occupant behaviour* sub-category looked at wood and biomass burning for heating and cooking as well as cookstove efficiency which are important determinants of poor household air quality and impact on a wide array of health endpoints ([Bailey et al. 2019](#_ENREF_14); [Quansah et al. 2017](#_ENREF_100)); low indoor temperatures may also direct effect blood pressure, blood clotting and other pathophysiological changes ([Jevons et al. 2016](#_ENREF_68)). Three studies addressed issue of environmental equity in relation to exposure to emissions from domestic wood fuel burning ([Bailey et al. 2019](#_ENREF_14)), air pollution impacts from cook stove and biomass burning ([Katoto et al. 2019](#_ENREF_71)), and thermal control in homes of the elderly ([Jevons et al. 2016](#_ENREF_68)).

3.2.2 Dwelling condition

Eight studies were grouped in this category under: *home improvements* (6 studies); and *soundness* (2 studies). Of the six studies on *home improvements*, three focussed on energy efficiency retrofitting ([Ige et al. 2019](#_ENREF_65); [Pega and Wilson 2016](#_ENREF_97); [Naldzhiev, Mumovic, and Strlic 2020](#_ENREF_91); [Bailey et al. 2019](#_ENREF_14); [Fenwick, Macdonald, and Thomson 2013](#_ENREF_49); [Jevons et al. 2016](#_ENREF_68)), with one including interventions to reduce falls ([Carnemolla and Bridge 2020](#_ENREF_31)). The two *soundness* studies reported on injuries from fire hazards ([Senthilkumaran et al. 2019](#_ENREF_118); [Bailey et al. 2019](#_ENREF_14); [Fenwick, Macdonald, and Thomson 2013](#_ENREF_49); [Jevons et al. 2016](#_ENREF_68)). Of all studies in this category, only those relating to home improvements reported on multiple health impacts; in addition to physical health, three studies looked at mental health ([Garin et al. 2014](#_ENREF_50); [Ige et al. 2019](#_ENREF_65); [Fenwick, Macdonald, and Thomson 2013](#_ENREF_49)); one focused on the elderly ([Garin et al. 2014](#_ENREF_50)).

3.2.3 Dwelling design

Eleven studies were identified under this category, further sub-categorised into three groups: *green buildings* (3 studies) looking at green walls, green design and green standards; *building type* (6 studies) to do with high-rise, type of dwelling, shared facilities etc; and *outdoor space* (2 studies).The green *buildings studies* assessed a range of health outcomes, including communicable disease (associated with water systems, vector borne disease), allergens and air quality ([Allen et al. 2015](#_ENREF_8); [Houghton and Castillo-Salgado 2019](#_ENREF_63), [2017](#_ENREF_62)). The *building type studies* reported o*n* physical space and mental health and wellbeing ([Barros et al. 2019](#_ENREF_17); [Garin et al. 2014](#_ENREF_50)), the impact of accessible design on falls and mental health ([Cho et al. 2016](#_ENREF_36)) and how design can minimise risks of injury, particularly later in life ([Garin et al. 2014](#_ENREF_50)); sedentary lifestyles associated with apartment or duplex living ([Chastin et al. 2015](#_ENREF_35)); physical and mental health, and wellbeing ([Carrere et al. 2020](#_ENREF_32)); and shared sanitation’s impact on communicable disease ([Heijnen et al. 2014](#_ENREF_59)). Two studies looked at health impacts associated with a dwelling’s *outdoor space*, including one on the physical activity benefits of gardens and outdoor equipment for children ([Carlin et al. 2017](#_ENREF_29)) and one on stress and time spent gardening ([Kondo, Jacoby, and South 2018](#_ENREF_74)).

3.2.4 Summary of building results

All systematic reviews in this category reported on physical health non-communicable outcomes associated with respiratory health (derived from exposure to allergens and air pollution) such as asthma, allergic, cardio-vascular, blood pressure or thermoregulation conditions; and safety/physical injury from specific housing types (e.g. duplex living). Only four reviews reported on communicable disease risks associated with water systems and shared sanitation as determinants for vector borne disease. Compared to physical health, mental health outcomes were less reported (6 studies) and in relation to a dwelling condition (e.g. thermal comfort) and design (e.g. type of building). Some reviews focused on vulnerable groups such as children and the elderly (indoor environmental quality studies); the elderly (dwelling condition) and the disabled (dwelling design).

There was limited consideration of planetary health impacts (6 studies). Studies reported on

associated carbon emissions from traffic ([Vardoulakis et al. 2020](#_ENREF_138)) and from compensatory ventilation to address indoor air pollution and emissions from particular types of thermal insulation ([Naldzhiev, Mumovic, and Strlic 2020](#_ENREF_91)); greenhouse gas mitigation, flood risk management, and ecological impacts for wildlife ([Allen et al. 2015](#_ENREF_8); [Houghton and Castillo-Salgado 2017](#_ENREF_62), [2019](#_ENREF_63)); and energy efficiency and efficient use of resources arising from high density living ([Barros et al. 2019](#_ENREF_17)).

## 3.3 Neighbourhood determinants of health (80 reviews)

Eighty studies (64.5%) reported on various aspects of the neighbourhood, which we broadly define here as an urban area made of residential and other buildings, as well as the supporting infrastructure for everyday living and its resident community . These were organised under 5 broad categories: ***green and blue infrastructure*** (34 studies), looking at *greenery, water collection and waste*; ***physical conditions*** (16 studies), reporting on the effects of *soundscape* (i.e. noise from traffic), *urban* design (i.e. street layout, lighting, walkability); and neighbourhood *renewal*; ***transpor****t (14 studies)*, including *traffic exposure*, *travel mode* and *mobility* aspects; *access to local service*s (7 studies) such as *shops*, *healthcare* and *education*; and ***socio-economic conditions*** (9 studies), reporting on effects of *disadvantage*, *social capital* and *crime* on health outcomes (see Table 2).

[Table 2 – insert here; see end of this document and Supplementary Table 2]

3.3.1 Green and blue infrastructure

Thirty-four studies were concerned with the relationship between *green and blue infrastructure* and health, grouped under *greenery* (32 studies) regarding various green aspects of neighbourhoods including parks, greenspace, vegetation, trees etc; and *water collection and waste* (2 studies) reporting on sewerage systems and water borne pathogens ([Jung et al. 2017](#_ENREF_69)) and pathogens in floodwater and respiratory disease ([Ishaq et al. 2020](#_ENREF_66)).

Of the *greenery* studies, 12 considered the relationship with physical health impacts only, mainly in the area of respiratory and cardiovascular disease or general health impacts ([Browning and Lee 2017](#_ENREF_25); [Twohig-Bennett and Jones 2018](#_ENREF_132); [Parker and de Baro 2019](#_ENREF_95); [Rugel and Brauer 2020](#_ENREF_106)); and 15 studies also reported mental health outcomes. Several reviews focused on the health of particular population groups including older adults and mortality ([Rugel and Brauer 2020](#_ENREF_106); [Yuan et al. 2020](#_ENREF_145); [Rojas-Rueda et al. 2019](#_ENREF_104)); children and asthma ([Hartley et al. 2020](#_ENREF_57)); pregnancy outcomes ([Akaraci et al. 2020](#_ENREF_4); [Lee, Moon, et al. 2020](#_ENREF_78)) and early childhood ([Islam, Johnston, and Sly 2020](#_ENREF_67)). Three studies specifically focused on the benefits of physical activity for health more generally ([Macmillan et al. 2018](#_ENREF_83)), children and adults with disabilities ([Saitta et al. 2019](#_ENREF_108)), and older adults ([Chastin et al. 2015](#_ENREF_35)). Five studies assessed various mental health outcomes including stress and anxiety more generally ([Calogiuri and Chroni 2014](#_ENREF_28); [Felappi et al. 2020](#_ENREF_48); [Gascon et al. 2017](#_ENREF_52); [Kondo et al. 2018](#_ENREF_73); [Chastin et al. 2015](#_ENREF_35)) and in children and adolescents ([Vanaken and Danckaerts 2018](#_ENREF_137)). One study assessed the mediating effects of vegetation on the relationship between stress and noise ([Dzhambov and Dimitrova 2018](#_ENREF_46)). Six studies reported other types of outcomes in addition to health including benefits to the economy and society ([Venkataramanan et al. 2019](#_ENREF_140)), social capital ([Venkataramanan et al. 2019](#_ENREF_140)), cognitive development in childhood and cognitive function in adulthood ([de Keijzer et al. 2016](#_ENREF_41)), cognitive function in children, adults and the elderly ([de Keijzer, Bauwelinck, and Dadvand 2020](#_ENREF_40)); environmental and health inequalities ([Schüle et al. 2019](#_ENREF_115)); and food security and nutrition outcomes ([Audate et al. 2019](#_ENREF_11)). The relationship between noise and green space was investigated in one study ([Dzhambov and Dimitrova 2018](#_ENREF_46)).

3.3.2 Physical conditions

Sixteen studies were grouped under this category considering *soundscapes* from road traffic mainly (6 studies); aspects of *urban design* (6 studies) and *neighbourhood renewal* (2 studies). Of these, six reviews examined the relationship between *soundscape* and health impacts including noise associations with hypertension ([Dzhambov and Dimitrova 2018](#_ENREF_46); [Van Kempen and Babisch 2012](#_ENREF_136)) and myocardial infarction ([Khosravipour and Khanlari 2020](#_ENREF_72)); pathways to health from noise ([Peris and Fenech 2020](#_ENREF_99)); noise levels, stress and self-reported general health ([Aletta, Oberman, and Kang 2018](#_ENREF_6))) and excessive noise and disability in later life ([Garin et al. 2014](#_ENREF_50)). A further 8 studies looked at different aspects of *urban design*: half of these were studies of physical activity and walkability, specifically looking at disabled people ([Eisenberg, Vanderbom, and Vasudevan 2017](#_ENREF_47)), successful aging ([Garin et al. 2014](#_ENREF_50)), the elderly ([Moran et al. 2014](#_ENREF_90); [Chastin et al. 2015](#_ENREF_35)) and three studies looked at physical health impacts associated with obesity-related outcomes such as type 2 diabetes and hypertension ([Chandrabose et al. 2019](#_ENREF_33); [Malambo et al. 2016](#_ENREF_84); [Schüle and Bolte 2015](#_ENREF_114)), one of which also considered mental health impacts ([Schüle and Bolte 2015](#_ENREF_114)) and one cardiometabolic risk ([Leal and Chaix 2011](#_ENREF_76)). Two studies reported on health outcomes associated with *neighbourhood renewal*; they focused on health impacts of housing regeneration schemes and socio-economic determinants of health ([Thomson and Thomas 2015](#_ENREF_127)) and impacts on mental and wellbeing from improvements in neighbourhood infrastructure such as access to transport and street greening ([Moore et al. 2018](#_ENREF_89)).

3.3.3 Transport

This category encompasses 14 studies grouped in three sub-categories: *traffic exposure* (6 studies)*, travel mode* (6 studies), and *mobility* (2 studies). Studies looking at *traffic exposure* reported on cardiovascular outcomes ([Malambo et al. 2016](#_ENREF_84)) in addition to mortality and other physical health impacts ([Rugel and Brauer 2020](#_ENREF_106)); lung cancer ([Hamra et al. 2015](#_ENREF_56)); and children and lung function ([Boothe et al. 2014](#_ENREF_24)), leukemia ([Barone-Adesi et al. 2015](#_ENREF_16)) and obesity ([Audrey and Batista-Ferrer 2015](#_ENREF_12)). Six studies reported on impacts of *travel mode* on health. Three studies were specifically concerned with cycling interventions, two looked at the potential for general public health impacts ([Stewart, Anokye, and Pokhrel 2015](#_ENREF_124); [Stankov et al. 2020](#_ENREF_123)) and one at physical activity and diet related health outcomes ([Macmillan et al. 2018](#_ENREF_83)). One study considered public transport and weight related health outcomes ([Patterson et al. 2019](#_ENREF_96)). The remaining two studies looked at transport costs and health impacts ([Möller et al. 2020](#_ENREF_88)) and mode of travel and health impacts in children from lower socio-economic groups ([Ma et al. 2020](#_ENREF_82)).Two reviews looked at transport *mobility* issues such as barriers to transport arising from congenital cardiovascular conditions in children and adults ([Davey et al. 2020](#_ENREF_39)) and transport mobility restrictions and health impacts including premature mortality in older adults ([Rosso, Auchincloss, and Michael 2011](#_ENREF_105)).

3.3.4 Access to local services

Seven studies were grouped in this category. Five studies focused specifically on access to and availability of local shops, with four looked at grocery shops and local food outlets such as supermarkets, farmer’s markets, restaurants and community kitchens ([Abeykoon, Engler-Stringer, and Muhajarine 2017](#_ENREF_1); [Macmillan et al. 2018](#_ENREF_83); [Malambo et al. 2016](#_ENREF_84); [Iacovou et al. 2013](#_ENREF_64)), and one at retail more generally ([Garin et al. 2014](#_ENREF_50)). In terms of health impacts, one study looked at access to healthcare and congenital heart disease in children and adults ([Davey et al. 2020](#_ENREF_39)) and another reported on the health of children from lower socio-economic groups and exposure to traffic from route to school ([Ma et al. 2020](#_ENREF_82)). Two studies considered BMI related outcomes in relation to grocery stores and supermarkets ([Abeykoon, Engler-Stringer, and Muhajarine 2017](#_ENREF_1); [Malambo et al. 2016](#_ENREF_84)), one of which also considered blood pressure, diabetes mellitus and metabolic syndrome associated with fast-food restaurants ([Malambo et al. 2016](#_ENREF_84)). Two studies reported on mental health outcomes: one looked at depression in the elderly and retail availability in the neighbourhood ([Garin et al. 2014](#_ENREF_50)); and another looked at self-reported health and psychological health and grocery shops ([Abeykoon, Engler-Stringer, and Muhajarine 2017](#_ENREF_1)). One study linked community kitchens with wellbeing benefits such as social engagement and community cohesion ([Iacovou et al. 2013](#_ENREF_64)).

3.3.5 Socio-economic conditions

The socio-economic characteristics of neighbourhoods impact health as they are underlying factors of: disadvantage and competition for scarce resources among neighbours; trust, social capital and collective action which can overcome challenges; and ‘contagious’ or ‘epidemic’ behaviours which makes neighbours to engage in similar behaviours ([Smelser and Baltes 2001](#_ENREF_122)). Nine studies were identified in this category reporting on *disadvantage* (3 studies);

*social capital* (4 studies); and *risky behaviours* (2 studies).

While only two studies reported on general physical health outcomes related to gentrification ([Bhavsar, Kumar, and Richman 2020](#_ENREF_22)) and teen pregnancy ([Smelser and Baltes 2001](#_ENREF_122)), the overall focus in this category and touched upon by all studies was on mental health conditions (e.g. depression, anxiety, self-reported health etc - 5 studies), health-risk behaviour (e.g. smoking, physical inactivity and early sex initiation -2 studies) and wellbeing (e.g. social health, loneliness - 4 studies) outcomes. Moreover, many studies reported health impacts on vulnerable groups such children ([Bhavsar, Kumar, and Richman 2020](#_ENREF_22); [Vyncke et al. 2013](#_ENREF_141); [Decker et al. 2018](#_ENREF_42)), ethnic minority populations ([Bécares, Dewey, and Das-Munshi 2018](#_ENREF_19); [Bhavsar, Kumar, and Richman 2020](#_ENREF_22)) and lower socio-economic groups ([Bhavsar, Kumar, and Richman 2020](#_ENREF_22)). One paper reported health impacts on all these three groups and also referred to planetary health outcomes in relation to environmental equity aspects of ‘green gentrification’ which can result in displacing vulnerable residents and augment their need for more emergency room or mental health visits as well as their food insecurity ([Bhavsar, Kumar, and Richman 2020](#_ENREF_22)).

The impact of *disadvantage* on health was reported in relation to neighbourhood deprivation and health-risk behaviour such as smoking and physical inactivity ([Algren et al. 2015](#_ENREF_7)); ethic segregation and mental health outcomes such as depression, anxiety, suicidality and suicide, psychotic experiences, and schizophreniform/psychotic disorders ([Smelser and Baltes 2001](#_ENREF_122)); and gentrification and self-reported health, physical and mental health outcomes and health-related behaviour, with a specific focus on negative health outcomes for ethnic groups, children and displaced residents ([Bhavsar, Kumar, and Richman 2020](#_ENREF_22)). Four studies reported on *social capital* related health outcomes looking at the amount of social capital in the neighbourhood and health outcomes in children and adolescents ([Vyncke et al. 2013](#_ENREF_141)); benefits of spending time spent others and mental health, quality of life and social health ([Lee, Burgess, et al. 2020](#_ENREF_77)); social cognition from leisure-time and health outcomes ([Rhodes, Saelens, and Sauvage-Mar 2018](#_ENREF_102)); and community life and healthy weight and depression ([Pérez et al. 2020](#_ENREF_98)). Two studies reporting on *risky behaviours* looked at unsafe local environments, early sexual initiation and adolescent pregnancy ([Decker et al. 2018](#_ENREF_42)), fear of crime and mental health ([Lorenc et al. 2013](#_ENREF_81)).

3.3.6 Summary of neighbourhood results

Across all reviews in this group three overall findings were apparent: (1) the *green and blue infrastructure* category received the most attention to date (34 studies); (2) physical health outcomes (respiratory and cardiovascular) were predominantly reported; however, mental health outcomes were also reported by 27 studies, especially in relation to greenspace (15 studies) and a neighbourhood’s socio-economic conditions (9 studies); interestingly, no transport study reported mental health outcomes; and (3) in contrast to the building level/group, nine studies reported on the socio-economic determinants of health and discussed these in relation to various vulnerable groups including children (15 studies), elderly (10 studies), disabled people (3 studies), women (3 studies), low income (2 studies) and ethnic minority groups (2 studies); the intersectionality of health outcomes was also considered in 3 studies reporting on adverse health outcomes on low-income and children or women.

One fifth of studies (16) reported on planetary health aspects, predominantly under the *greenery* category (9 studies). Reported aspects included biodiversity ([Lai et al. 2019](#_ENREF_75)), (cultural) ecosystem services ([Zhang et al. 2017](#_ENREF_146)), conflicts between wildlife and human needs ([Felappi et al. 2020](#_ENREF_48)), environmental resources ([Schüle et al. 2019](#_ENREF_115)), environmental benefits ([Parker and de Baro 2019](#_ENREF_95)) and air pollution ([Lee, Moon, et al. 2020](#_ENREF_78)), sustainability related aspects such as flooding ([Ishaq et al. 2020](#_ENREF_66); [Venkataramanan et al. 2019](#_ENREF_140)) and the SDGs ([Vanaken and Danckaerts 2018](#_ENREF_137)); mediating effects of weather and environmental conditions on physical activity in older adults ([Moran et al. 2014](#_ENREF_90)); carbon emissions from road traffic ([Barone-Adesi et al. 2015](#_ENREF_16); [Hamra et al. 2015](#_ENREF_56); [Rugel and Brauer 2020](#_ENREF_106)); and air pollution from transport ([Möller et al. 2020](#_ENREF_88)) ([Ma et al. 2020](#_ENREF_82)).

## 3.4 Urban system determinants of health (32 reviews)

Local built environments are part of the wider urban system which extends beyond buildings and neighbourhoods, to the whole city, immediate but also distant built or un-built hinterlands of regions, nations and, ultimately, to the planet. These are parts of the urban system, connected by complex relations and feedback loops, which in turn can influence outcomes at the local level. In the housing sector, this occurs through, but it is not limited to, (i) planned action of housing at scale via *spatial planning*; (ii) the unplanned, albeit regulated interactions of agents and institutions seeking and providing housing represented by the *housing system*; and (iii) via the consequential desired and undesired impacts on natural *ecosystems*. Thirty-two studies (25.8%) reported on urban system determinants of health categorised as *spatial planning* (17 studies); *housing system* (11 studies): *ecosystems* (4 studies) (see Table 3).

[Table 3 – insert here; see end of this document and Supplementary Table 3]

3.4.1 Spatial planning

Seventeen studies were identified in this category reporting on health outcomes and *informality*  (3 studies), *urban infrastructure* (3 studies), *type of development* (3 studies) and *masterplanning* (8 studies). The three studies reporting on *informality* assessed the health impacts of strategies to improve the infrastructure, conditions and land tenure of slums, including communicable and non-communicable disease prevention, the risk of injury from chemical and biological hazards, as well as social impacts such as quality of life, education and employment ([Alaazi and Aganah 2020](#_ENREF_5); [Turley et al. 2013](#_ENREF_131); [Henson et al. 2020](#_ENREF_60)). Three studies looked at *urban infrastructure*: one study assessed urban drinking water and gastroenteritis risk ([Beaudeau 2018](#_ENREF_18)); one study reported on the structural soundness of the city in the face of earthquakes and subsequent building collapses ([Doocy et al. 2013](#_ENREF_43)); and one study looked at urban exposure to overhead powerlines ([Habash et al. 2019](#_ENREF_55)). *Type of development* effects were reported in three studies, in relation to walkability, physical activity behaviours and obesogenic health impacts ([Berghauser Pont et al. 2020](#_ENREF_21); [Chandrabose et al. 2019](#_ENREF_33); [Cyril, Oldroyd, and Renzaho 2013](#_ENREF_38)). Eight studies focussed on aspects of *masterplanning* at the city level and health outcomes such as morbidity and mortality related to non-communicable disease risks (i.e. physical inactivity), injury and mental health. More specifically, these studies focused on nature-base approaches ([Kabisch, van den Bosch, and Lafortezza 2017](#_ENREF_70)), age-friendly infrastructure for the elderly or children ([Sánchez-González et al. 2020](#_ENREF_111); [Nordbø et al. 2018](#_ENREF_92)) and smart city technologies ([Rocha et al. 2019](#_ENREF_103)). Strategic planning and smart growth approaches were also included ([McCrorie, Fenton, and Ellaway 2014](#_ENREF_87); [Durand et al. 2011](#_ENREF_45); [Gong et al. 2016](#_ENREF_54); [Salgado et al. 2020](#_ENREF_109)).

3.4.2 Housing system

Housing system studies were 11 and categorised into two categories: *vulnerability* (8 studies), and *policy* (3 studies). In the *vulnerability* sub-category, three studies looked at how foreclosure, either directly experienced or general risk in the neighbourhood, negatively affected physical and mental health, as well as health-relevant behaviours (including substance misuse and violence) ([Downing 2016](#_ENREF_44); [Tsai 2015](#_ENREF_128); [Vásquez-Vera et al. 2017](#_ENREF_139)); one study looked at how combinations of tenure precarity and poor physical characteristics of dwellings may combine to influence mental health ([Singh et al. 2019](#_ENREF_121)); one study assessed how permanent supportive social housing may benefit both health (e.g. mental health, hospital admissions) and health-supporting social conditions (e.g. employment and income) ([Aubry et al. 2020](#_ENREF_10)); and three studies looked at vulnerability by specifically focussing on a population subgroup (people living with HIV) ([Aidala et al. 2016](#_ENREF_2)), a risk type (cold weather) ([Tanner et al. 2013](#_ENREF_126)), and an outcome (congenital heart disease) ([Davey et al. 2020](#_ENREF_39)). Of the three studies classified in the *policy* sub-category, two looked at how a range of material housing changes and support contributed to changes in the risk of obesity ([Tseng et al. 2018](#_ENREF_129)) and diabetes ([Barnard et al. 2015](#_ENREF_15)). A third study considered strategies that combined material (e.g. built infrastructure) and social (e.g. community networking and empowerment) aspects to create healthy environments (e.g. availability of healthy food; encouragement of physical activity) that would in turn improve community health ([Chaparro et al. 2020](#_ENREF_34)).

3.4.3 Natural ecosystems

Four studies reported on natural *ecosystems* such as *air, water* and *climate*. Two studies considered air pollution and its effects on cardiorespiratory outcomes in people of different age groups in Sub-Saharan Africa ([Katoto et al. 2019](#_ENREF_71)), and the health and health equity benefits of interventions aiming to reduce air pollution levels ([Benmarhnia et al. 2014](#_ENREF_20)). One study assessed the mental health benefits of wider natural ecosystems for residential areas ([Gascon et al. 2017](#_ENREF_52)), and one paper looked at microclimate influences on urban heat islands and the resulting impacts of mortality and cardiorespiratory morbidity ([Schinasi, Benmarhnia, and De Roos 2018](#_ENREF_112)).

3.4.4 Summary of urban system results

More than half (17 studies) of all studies in this group focused on the impact of *spatial planning* on both health exposure and health interventions. Physical health impacts were discussed in relation to both communicable and non-communicable disease, more specifically general health outcomes, mortality and morbidity, walkability and physical activity outcomes, drinking water quality and communicable disease, resilience to natural disasters, cancer, reproductive health, obesity and cardiorespiratory. Mental health impacts were discussed in relation to *informality* and *masterplanning* aspects; *housing system*’s vulnerability (mainly measures of housing insecurity) and policies (material change and material and social support) aspects, and the natural *ecosystem*. There was some evidence reporting on the health of particular demographic groups such as the elderly and children (under *spatial planning* ), and vulnerable groups including those with underlying health conditions (HIV, heart disease) and especially those with insecure housing tenure (under *housing system*).

Surprisingly, less than one sixth of studies (5) discussed planetary health outcomes. These reported on the impacts of rapid urbanization on the environment and implications for the SDGs ([Henson et al. 2020](#_ENREF_60)) ([Chandrabose et al. 2019](#_ENREF_33)); climate change impacts of air pollution ([Benmarhnia et al. 2014](#_ENREF_20); [Katoto et al. 2019](#_ENREF_71)) and microclimates ([Schinasi, Benmarhnia, and De Roos 2018](#_ENREF_112)).

# Discussion

This paper provides an overview of the last decade’s evidence on health and urban housing from a spatial scaleperspective. Most of the systematic review evidence (64.5%) reviewed by this paper focussed on the *neighbourhood* level of the local built environment; while the *building* and *urban system* levels accounted for the rest in equal shares. Across the three scales, however, 5 out of 11 categories of built environment determinants of health have received most attention to date: *green and blue structure* (32/124); *spatial planning* (17/124), neighbourhood’s *physical conditions* (16/124) and *transport* (14/124), and dwelling’s *indoor environmental quality* (12/124). The number of reviews focusing on health impacts at the building level was less than anticipated; this can be explained by the fact that existing evidence is not published in English yet and/or summarised by systematic reviews during 2011-2020.

Three overall observations can be made across all scales and all studies. Physical health outcomes remained predominantly reported by systematic reviews, primarily in relation to non-communicable disease (NCDs) in high income settings. This may be because the majority of reviews focused on developed contexts where NCDs form the predominant burden of health. Mental health outcomes were included in 40 studies (1/3 of all studies) 27 were at the neighbourhood level, 6 and 7 at the building and urban system scale, respectively. Only one fifth of rstudies (27/124) reported some planetary health outcomes, and again mostly at the neighbourhood level (16/27), 6/27 at building and 5/27 at urban system level.

## 4.1 Where next for research

This umbrella review indicates three potential directions for further research, more generally, and systematic review research, more specifically. First, research on urban health usually involves two distinct communities of scholars, health scientists and urban scientists, who can come from completely different research paradigms ([Turcu et al. 2021](#_ENREF_130)). This requires time to learn or synthetise across disciplines, transdisciplinary methods to account for the variety of entry points, but also relational thinking to acknowledge the multiple connections between the different elements of the urban system and the continuum of health outcomes e.g. physical – mental - wellbeing. The studies identified by this paper come from teams of primarily health scientists hence, grounding findings with urban scientists can be challenging. There is also a predominant focus on the ‘negative psychology’ approach to health whereby treating the effects of a particular condition (i.e. cardiovascular, respiratory, BMI, blood pressure) is in focus, as opposed to ‘positive psychology’ approach when the cause of the condition (poverty/ deprivation, vulnerability) is analysed ([Seligman 2004](#_ENREF_116)). While some mental health and wellbeing outcomes are mentioned at the neighbourhood and wider urban system level, they need better understanding. Here, a ‘flourish’ approach to health can be explored, whereby the focus is on people, rather than their health, under the assumption that improving wider quality of life and social health would make for healthier people ([Seligman 2011](#_ENREF_117)).

Second, there are three obvious gaps in the literature: the intersectionality of health outcomes, lack of evidence from low-income settings, and little current discussion of communicable disease burden. While there are often clear pathways for increased risk for vulnerable population groups, review evidence for these groups appears to be limited and tends to be focused on children and the elderly; there is little consideration of how, for example, age, gender and socio-economic status may intersect in the built environment and affect health. There was also very limited evidence from low-income countries and/or communicable disease outcomes; where reviews were identified they reported health outcomes associated with indoor air pollution from cooking stoves and informal living, lack of water infrastructure and sanitation, and vector borne disease. The current COVID-19 pandemic will certainly through the focus back onto communicable disease outcomes.

Third, planetary health impacts are mainly reported in relation to carbon emissions. Expanding understanding beyond this point is another research direction worth exploring. Research reporting on planetary impacts in conjunction with human health ones, across all scales of the built environment can reinforce advocacy for urban sustainability transitions. If the challenges of the health-climate crisis is to be met, the nexus of human and planetary health needs better understanding of unintended consequences, better policy making and urban governance at all levels ([Crane et al. 2021](#_ENREF_37)).

## 4.2 A role for policy and urban governance

Urban health research is closely associated with relevance to policymaking ([Hawkes et al. 2016](#_ENREF_58); [Sallis et al. 2016](#_ENREF_110); [Schneidera and Blythb 2017](#_ENREF_113); [Turcu et al. 2021](#_ENREF_130)). This paper found that many studies note implications for policymaking and urban stakeholders (e.g. urban planners, landscape architects, communities, residents etc), but discussion is rather general. This may be explained by the fact that most reviews take a health perspective whereby roles outside health in implementing or changing exposure risks in the built environment are not considered. In what follows, this paper contributes to expanding policy understanding in this area by exploring different types of policy interventions and actors involved across the scales of the built environment.

At the *building* level, exposure to many identified environmental risk factors are long-term and difficult to modify without substantial investment of time and resources. For example, remedial factors to address risks from exposure to low indoor temperatures via energy retrofitting to protect against winter cold may take substantial investment. Likewise, factors related to a building’s condition have clear benefits in terms of health when considering safety measure (e.g. injury from fire, falls etc) and building regulations usually address these issues, but regulation and compliance may be challenging in some settings or differ amongst sectors and professions. The building level is usually addressed by architects, designers, developers, building contractors, owners of individual structures and health practitioners.

At the *neighbourhood* level, the weight of evidence suggests positive health outcomes are associated with green space, although these may be confounded by socio-economic status i.e. wealthier neighbourhoods having higher density of green space and living in deprived/ poor neighbourhoods is linked to adverse health impacts for vulnerable groups such as children, the elderly and disable people, women and ethnic minority groups. While it may be challenging to add new green space to established cities, modifications to existing green space can be made to encourage physical activity, along with fair and equal access for all socio-economic groups and education to effect behaviour change. Actors involved in the governance of the neighbourhood level primarily include local government, local planning and local health trusts, communities, civil society and business organisations.

At the *urban system* level, the evidence presented points to at least two important ‘alignments’ e.g. between health, spatial planning and housing policy; and between climate change and health outcomes. City level spatial planning policies can impact on health by providing adequate levels of affordable or social housing and so, de-risking housing security, an important socio-economic determinant of health, while housing policy at the regional or national level can help to absorb shock-related health impacts from events such as the financial crisis in 2007-2008 or current COVID-19 pandemic. Furthermore, increasing greenspace and energy retrofitting are associated with clear health and climate change positive outcomes; high density or shared living can reduce pressure on resources and associated carbon emissions, but some evidence suggests mental health negative outcomes from overcrowding and impacts on physical health from limited space. The latter is easier to address in policy terms – for example, provision of easily accessible parks and recreational facilities to allow physical activity, while overcrowding is harder to address and requires long term action and investment, but policy measures such as standards and regulations can help. The governance of the urban system involves all the actors at the building and neighbourhood level and much more i.e. regional and national government involved in strategic policymaking, but also global organisations including international institutions such as the WHO and EU.

The examples above frame urban health within the wider process of policymaking in the build environment. Policymaking is a complex and, most importantly, a political process; it is not something happening at a particular time, in a particular spot, but part of wider multi-level governance frameworks, which frame the complexity of the urban system and local built environment. As seen above, the governance of urban health involves policymakers responsible for health- or housing-related policy and regulation and other stakeholders involved in health interventions such as government agencies, architects, builders, housing providers, developers, engineers, urban planners, industry regulators, financial institutions, as well as social services, community groups, and public health professionals. These stakeholders are ultimately required to ensure that housing is built, maintained, renovated, used and demolished in ways that support health.

By taking a scale perspective on health, the connections between different policies and levels of policymaking become apparent. For example, national government needs to align health with SDGs, ensure geographic equity and combat siloed approaches, while local government must ensure that public health and spatial planning work together to strengthen the link between people and places and break administrative boundaries to reap the benefits of planetary health. Also, the wider the scale the more complex the array of actors involved and the dynamic lines of power and networks inside and outside policymaking ([Bulkeley and Kern 2006](#_ENREF_27)). Communities may hold important knowledge about public health in their locality, but may not have a voice, especially if marginalised; civil society organisations may lobby or support government and communities, while professional communities such as urban planners share many communalities with public health professionals including an interest in the public good, and use of evidence-based and long-term-assessment approaches ([WHO 2020](#_ENREF_144)). If evidence fails to engage with the multi-scalar multi-level nature of urban governance, which makes links across scales from local to supranational and where power is distributed across horizontal and vertical networks which do not operate in a hierarchical manner ([Bulkeley and Betsill 2005](#_ENREF_26)). This paper suggests that a better grounding of urban health research in existing urban governance landscapes, would not only support a faster and more efficient implementation of health interventions at the local level, but also gauge potential synergies and tensions at the urban system level with other pressing urban challenges such as climate change.

## 4.3 Strengths and limitations

This paper is novel for synthesising evidence on housing from the perspective of scales within the urban built environment. Most research has either considered housing from a narrow understanding of the individual’s dwelling or disconnected the built environment from its purpose of providing liveability ([Giles-Corti, Lowe, and Arundel 2019](#_ENREF_53)). To our knowledge, this is the first umbrella review which takes a comprehensive view of local built environment and housing at scale, while looking at both physical and socio-economic characteristics that define the complex urban system.

This review is limited to systematic review evidence and generic geographical regions. Evidence derived purposely for specific countries were excluded because of the specific content and context. As such we acknowledge the broad conclusions made in this review. The intent was not to assess the size of health impacts or the effectiveness of interventions and make no assessment of the importance of one health determinant or built factor over another. Furthermore, the study excluded research on shelters for the homeless, residential care or student accommodation, rural housing, which also pose built environment, societal and health concerns.

# Conclusion

This paper provides an umbrella review of reported health impacts across three broad spatial scales and notes that most research has focused on health at the neighbourhood level. Discussions of planetary health and policy implications have been limited, and only a few studies have evaluated the economic implications of health interventions. This study is the first of its type: it applies a scale perspective to health, suggests directions for potential future research and expands the discussion of urban governance for health.

**Funding information**

This research was funded in whole, or in part, by the Wellcome Trust [Grant number 209387/Z/17/Z]. **For the purpose of Open Access, the author has applied a CC BY public copyright licence to any Author Accepted Manuscript version arising from this submission.**

**Competing interests**

Nothing to declare

**Data availability**

Not applicable

**Authors’ contributions**

CT conceived the idea for the paper, drafted the manuscript and dealt with the paper revisions. MT and CT designed the methodology and compiled the search and library of data. CT, MC, EH, KB and SL screened the papers. All authors contributed to the writing of the paper.

Table 1 – Systematic reviews reporting on BUILDING determinants of health

| ***Determinant*** | ***Specific aspects*** | ***Human health*** | ***Planetary health*** |
| --- | --- | --- | --- |
| **Indoor environmental quality** | **Air quality**  (e.g. particles including radon, fungi, PM, NO2, VOC; and, chemicals) | Impacts of exposure on lung cancer ([Ajrouche et al. 2017](#_ENREF_3)); cognitive and neurobehavioral outcomes ([Nussbaumer-Streit et al. 2020](#_ENREF_94)); asthma exacerbation ([Sharpe et al. 2015](#_ENREF_119)) ([Tagiyeva and Sheikh 2014](#_ENREF_125)) ([Naldzhiev, Mumovic, and Strlic 2020](#_ENREF_91)) ([Van Boven et al. 2020](#_ENREF_134)); respiratory and general health & wellbeing ([Vardoulakis et al. 2020](#_ENREF_138)). | Air pollution (PM, NO2, VOCs etc) ([Vardoulakis et al. 2020](#_ENREF_138)); energy demand ([Naldzhiev, Mumovic, and Strlic 2020](#_ENREF_91)). |
|  | **Occupant hazards**  (e.g. burning fuel, ventilation, room temperature) | Impacts of exposure on thermal control in the elderly and those with respiratory conditions ([Jevons et al. 2016](#_ENREF_68)); cardio-respiratory, paediatric, maternal outcomes and mortality ([Lee, Bing, et al. 2020](#_ENREF_79)) ([Katoto et al. 2019](#_ENREF_71)).  Interventions to reduce exposure and general health benefits to children and adults. ([Quansah et al. 2017](#_ENREF_100); [Bailey et al. 2019](#_ENREF_14)) | Environmental equity ([Bailey et al. 2019](#_ENREF_14)); air pollution (PM) ([Katoto et al. 2019](#_ENREF_71)) ([Quansah et al. 2017](#_ENREF_100)); climate temperature ([Jevons et al. 2016](#_ENREF_68)). |
| **Dwelling condition** | **Home improvements**  (e.g. energy retrofit, upgrade of bathrooms and kitchens) | Injury and falls prevention, mobility, independence and wellbeing ([Carnemolla and Bridge 2020](#_ENREF_31)); health economic benefits ([Fenwick, Macdonald, and Thomson 2013](#_ENREF_49)); reduction in depression in the elderly ([Garin et al. 2014](#_ENREF_50)); respiratory outcomes, QoL and mental health ([Ige et al. 2019](#_ENREF_65)); lung disease prevention ([Pega and Wilson 2016](#_ENREF_97)) | Energy required for ventilation ([Naldzhiev, Mumovic, and Strlic 2020](#_ENREF_91)). |
|  | **Soundness**  (e.g. risk of fire, falls, structural integrity) | Interventions to reduce Fire-related deaths and injuries avoided ([Senthilkumaran et al. 2019](#_ENREF_118)) ([Pega and Wilson 2016](#_ENREF_97)). |  |
| **Dwelling design** | **Green buildings**  (e.g. green designs, green standards, green walls) | Impacts of interventions on: respiratory symptoms and general wellbeing ([Allen et al. 2015](#_ENREF_8)); flood-risk and outcomes such as waterborne diseases, mortality, and psychological harm ([Houghton and Castillo-Salgado 2019](#_ENREF_63)); heat-related morbidity and mortality ([Houghton and Castillo-Salgado 2017](#_ENREF_62)). | Reduced energy use and C02 emissions ([Allen et al. 2015](#_ENREF_8)); attention to the interface between humans, habitats, wildlife, and water systems ([Houghton and Castillo-Salgado 2019](#_ENREF_63)) ([Houghton and Castillo-Salgado 2017](#_ENREF_62)). |
|  | **Building type**  (e.g. high-rise, apartment, duplex, co-housing, accessible-by-design, size, shared sanitation) | Impacts of conditions on: social well-being, QoL, and mental health ([Barros et al. 2019](#_ENREF_17)) ([Carrere et al. 2020](#_ENREF_32)) ([Cho et al. 2016](#_ENREF_36)) ([Garin et al. 2014](#_ENREF_50)); sedentary lifestyles ([Chastin et al. 2015](#_ENREF_35)); falls and mortality ([Cho et al. 2016](#_ENREF_36)); infectious diseases and maternal outcomes ([Heijnen et al. 2014](#_ENREF_59)). | Efficient use of resources ([Barros et al. 2019](#_ENREF_17)). |
|  | **Outdoor space**  (e.g. gardens, outdoor equipment) | Interventions to improve physical activity of children ([Carlin et al. 2017](#_ENREF_29)) , and stress reduction (e.g HR and BP) ([Kondo, Jacoby, and South 2018](#_ENREF_74)). |  |

*Legend: BP (Blood pressure), CO2 (Carbon dioxide), HR (heart rate), NO2 (Nitrogen Dioxide), PM (particulate matter), QoL (quality of life), VOC (Volatile Organic Compounds).*

Table 2 – Systematic reviews reporting on NEIGHBOURHOOD determinants of health

| ***Determinant*** | ***Specific aspects*** | ***Human health*** | ***Planetary health*** |
| --- | --- | --- | --- |
| **Green and blue infrastructure** | **Greenery**  (e.g. green- and natural space, contact with nature, green infrastructure, urban agriculture) | Impacts on: physical, mental, and/or social health and mortality ([Browning and Lee 2017](#_ENREF_25)) ([Calogiuri and Chroni 2014](#_ENREF_28)) ([Carmona 2019](#_ENREF_30)) ([Venkataramanan et al. 2019](#_ENREF_140)) ([van den Berg et al. 2015](#_ENREF_135)) ([de Keijzer, Bauwelinck, and Dadvand 2020](#_ENREF_40)) ([Dzhambov and Dimitrova 2018](#_ENREF_46)) ([Felappi et al. 2020](#_ENREF_48)) ([Shuvo et al. 2020](#_ENREF_120)) ([Gascon et al. 2015](#_ENREF_51)) ([Kondo et al. 2018](#_ENREF_73)) ([Lai et al. 2019](#_ENREF_75)) ([Rojas-Rueda et al. 2019](#_ENREF_104)) ([Parker and de Baro 2019](#_ENREF_95)) ([Rugel and Brauer 2020](#_ENREF_106)) ([Macmillan et al. 2018](#_ENREF_83)) ([Audate et al. 2019](#_ENREF_11)), including for children ([Vanaken and Danckaerts 2018](#_ENREF_137)) ([de Keijzer et al. 2016](#_ENREF_41)) ([Hartley et al. 2020](#_ENREF_57)) ([Islam, Johnston, and Sly 2020](#_ENREF_67)) ([McCormick 2017](#_ENREF_86)), maternal health ([Twohig-Bennett and Jones 2018](#_ENREF_132)) ([Lee, Moon, et al. 2020](#_ENREF_78)), elderly people ([Yuan et al. 2020](#_ENREF_145)) ([Garin et al. 2014](#_ENREF_50)) ([Levy-Storms, Chen, and Loukaitou-Sideris 2018](#_ENREF_80)) ([Chastin et al. 2015](#_ENREF_35)), people with disabilities ([Zhang et al. 2017](#_ENREF_146)) ([Saitta et al. 2019](#_ENREF_108)), and health inequalities ([Schüle et al. 2019](#_ENREF_115)). | Ecosystem services, wildlife and biodiversity ([Zhang et al. 2017](#_ENREF_146)) ([Felappi et al. 2020](#_ENREF_48)) ([Schüle et al. 2019](#_ENREF_115)) ([Lai et al. 2019](#_ENREF_75)) ([Parker and de Baro 2019](#_ENREF_95)); floods ([Yuan et al. 2020](#_ENREF_145)); air pollution([Lee, Moon, et al. 2020](#_ENREF_78)) ; contributions to the SDGs ([Vanaken and Danckaerts 2018](#_ENREF_137)). |
|  | **Water collection and waste**  (e.g. blue space, urban drains, sewage) | Health risks associated with floods ([Ishaq et al. 2020](#_ENREF_66)) and diarrhoeal disease ([Jung et al. 2017](#_ENREF_69)). | Floods ([Ishaq et al. 2020](#_ENREF_66)). |
| **Physical conditions** | **Soundscape**  (e.g. noise, including from traffic; noise buffers) | Hypertension ([Van Kempen and Babisch 2012](#_ENREF_136)) ([Dzhambov and Dimitrova 2018](#_ENREF_46)), myocardial infarction ([Khosravipour and Khanlari 2020](#_ENREF_72)), stress-recovery and self-reported health ([Aletta, Oberman, and Kang 2018](#_ENREF_6)), disability in elderly people ([Garin et al. 2014](#_ENREF_50)), as well as combined pathways to health ([Peris and Fenech 2020](#_ENREF_99)). | Ecosystem responses to transport noise and natural environment impact on noise ([Peris and Fenech 2020](#_ENREF_99)). |
|  | **Urban design**  (e.g. walkability, rest areas and benches, street layout and connectivity) | Physical health including hypertension, BMI, and type 2 diabetes ([Chandrabose et al. 2019](#_ENREF_33)) ([Leal and Chaix 2011](#_ENREF_76)) ([Malambo et al. 2016](#_ENREF_84)); physical activity in elderly people ([Chastin et al. 2015](#_ENREF_35)) ([Moran et al. 2014](#_ENREF_90)) and people with disabilities ([Eisenberg, Vanderbom, and Vasudevan 2017](#_ENREF_47)); child accidents ([Schüle and Bolte 2015](#_ENREF_114)); mental health and QoL ([Schüle and Bolte 2015](#_ENREF_114)) ([Garin et al. 2014](#_ENREF_50)). | Weather and environmental conditions ([Moran et al. 2014](#_ENREF_90)). |
|  | **Neighbourhood renewal**  (e.g. improvement, upgrade, renewal) | Impacts on socioeconomic determinants of health ([Thomson and Thomas 2015](#_ENREF_127)), and mental health and wellbeing ([Moore et al. 2018](#_ENREF_89)). |  |
| **Transport** | **Traffic exposure**  (e.g. measures, street design) | Morbidity and mortality associated with cardiovascular, respiratory, metabolic, and reproductive health ([Hamra et al. 2015](#_ENREF_56)) ([Rugel and Brauer 2020](#_ENREF_106)) ([Malambo et al. 2016](#_ENREF_84)), including child health for lung function ([Barone-Adesi et al. 2015](#_ENREF_16)), leukaemia ([Boothe et al. 2014](#_ENREF_24)) and obesity ([Audrey and Batista-Ferrer 2015](#_ENREF_12)). | Air pollution ([Barone-Adesi et al. 2015](#_ENREF_16)) ([Hamra et al. 2015](#_ENREF_56)) ([Rugel and Brauer 2020](#_ENREF_106)). |
|  | **Travel mode**  (e.g. public transportation, cycling, walking) | Changes in cycling behaviour ([Stewart, Anokye, and Pokhrel 2015](#_ENREF_124)); health associated with physical activity, air pollution and injuries ([Stankov et al. 2020](#_ENREF_123)) ([Patterson et al. 2019](#_ENREF_96)) ([Möller et al. 2020](#_ENREF_88)) ([Macmillan et al. 2018](#_ENREF_83)); health of children from disadvantaged socioeconomic groups ([Ma et al. 2020](#_ENREF_82)). | Air pollution ([Möller et al. 2020](#_ENREF_88)) and environmental justice ([Ma et al. 2020](#_ENREF_82)) . |
|  | **Mobility**  (e.g. transport barriers and restrictions) | Premature mortality in elderly people ([Rosso, Auchincloss, and Michael 2011](#_ENREF_105)) and congenital heart disease ([Davey et al. 2020](#_ENREF_39)). |  |
| **Access to local services** | **Shops**  (e.g. grocery, supermarkets, farmer markets, community kitchen, retail) | Physical activity- and dietary-related health outcomes including blood pressure, BMI, type 2 diabetes, mental health, and self-reported health ([Abeykoon, Engler-Stringer, and Muhajarine 2017](#_ENREF_1)) ([Garin et al. 2014](#_ENREF_50)) ([Macmillan et al. 2018](#_ENREF_83)) ([Malambo et al. 2016](#_ENREF_84)) ([Iacovou et al. 2013](#_ENREF_64)). |  |
|  | **Healthcare**  (e.g. primary care) | Congenital heart disease ([Davey et al. 2020](#_ENREF_39)) . |  |
|  | **Education**  (e.g. schools) | Health of children from disadvantaged socioeconomic groups ([Ma et al. 2020](#_ENREF_82)). | Environmental justice ([Ma et al. 2020](#_ENREF_82)). |
| **Socio-economic conditions** | **Disadvantage**  (e.g. deprivation, segregation, gentrification) | Impacts on self-reported health, mental health and health-related behaviours ([Algren et al. 2015](#_ENREF_7)) ([Bécares, Dewey, and Das-Munshi 2018](#_ENREF_19)) ([Bhavsar, Kumar, and Richman 2020](#_ENREF_22)). | Environmental equity ([Bhavsar, Kumar, and Richman 2020](#_ENREF_22)). |
|  | **Social capital**  (e.g. time spent with others, leisure activities, social cohesion) | Physical activity, mental and social health([Lee, Burgess, et al. 2020](#_ENREF_77)) ([Rhodes, Saelens, and Sauvage-Mar 2018](#_ENREF_102)) ([Pérez et al. 2020](#_ENREF_98)), including benefits for children and adolescents ([Vyncke et al. 2013](#_ENREF_141)). |  |
|  | **Risk**  (e.g. unsafe environment, crime) | Earlier sexual initiation and increased adolescent pregnancy ([Decker et al. 2018](#_ENREF_42)); mental health risks due to fear of crime ([Lorenc et al. 2013](#_ENREF_81)). |  |

*Legend: BMI (body mass index), QoL (quality of life), SDG (Sustainability Development Goals), Urban Heat Islands (UHI).*

Table 3 – Systematic reviews reporting on URBAN SYSTEM determinants of health

| ***Determinant*** | ***Specific aspects*** | ***Human health*** | ***Planetary health*** |
| --- | --- | --- | --- |
| **Spatial planning** | **Informality**  (e.g. slums) | Physical and mental health, as well as QoL and social capital ([Alaazi and Aganah 2020](#_ENREF_5)) ([Turley et al. 2013](#_ENREF_131)) ([Henson et al. 2020](#_ENREF_60)). | Rapid urbanization and effects on the environment and SDGs ([Henson et al. 2020](#_ENREF_60)). |
|  | **Urban infrastructure**  (e.g. water, power lines, urban structure) | Morbidity and mortality – including gastroenteritis, cancer, CVD, reproductive outcomes, and neurogenerative disease – and risk of displacement following earthquakes ([Beaudeau 2018](#_ENREF_18)) ([Doocy et al. 2013](#_ENREF_43)) ([Habash et al. 2019](#_ENREF_55)). |  |
|  | **Type of development**  (e.g. densification, built form typology, urbanicity) | Health-related behaviours including physical activity and fruit and vegetable consumption, and associated outcomes such as obesity, cardiometabolic diseases and mental health ([Berghauser Pont et al. 2020](#_ENREF_21)) ([Chandrabose et al. 2019](#_ENREF_33)) ([Chandrabose et al. 2019](#_ENREF_33)). | Densification contributions to the SDGs ([Berghauser Pont et al. 2020](#_ENREF_21)). |
|  | **Masterplanning**  (e.g. urban designing for: active ageing, healthcare access, smart growth, smart city, landscaping) | Physical activity ([Nordbø et al. 2018](#_ENREF_92)) ([McCrorie, Fenton, and Ellaway 2014](#_ENREF_87)) ([Durand et al. 2011](#_ENREF_45)) ([Rocha et al. 2019](#_ENREF_103)), mental health ([Gong et al. 2016](#_ENREF_54)), including the health of children and the elderly ([Sánchez-González et al. 2020](#_ENREF_111)) ([Kabisch, van den Bosch, and Lafortezza 2017](#_ENREF_70)), and morbidity and mortality ([Salgado et al. 2020](#_ENREF_109)). | Air pollution, noise, UHI, green and blue space ([Salgado et al. 2020](#_ENREF_109)) ([Kabisch, van den Bosch, and Lafortezza 2017](#_ENREF_70)) ([Rocha et al. 2019](#_ENREF_103)). |
| **Housing system** | **Vulnerability**  (e.g. housing status, insecurity and instability; social housing) | Physical health, mental health, and health-related behaviours ([Vásquez-Vera et al. 2017](#_ENREF_139)) ([Downing 2016](#_ENREF_44)) ([Tsai 2015](#_ENREF_128)) ([Singh et al. 2019](#_ENREF_121)) ([Davey et al. 2020](#_ENREF_39)) ([Aidala et al. 2016](#_ENREF_2)), as well as health-related social outcomes ([Aubry et al. 2020](#_ENREF_10)) ([Tanner et al. 2013](#_ENREF_126)). | Energy access and fuel poverty ([Tanner et al. 2013](#_ENREF_126)). |
|  | **Policy**  (e.g. housing support, healthy municipality strategy) | Diabetes ([Singh et al. 2019](#_ENREF_121)), obesity ([Tseng et al. 2018](#_ENREF_129)), and community health status ([Chaparro et al. 2020](#_ENREF_34)). |  |
| **Ecosystems** | **Air**  (e.g. pollution) | Mortality, cardiorespiratory health, and health equity ([Benmarhnia et al. 2014](#_ENREF_20)) ([Katoto et al. 2019](#_ENREF_71)). | Air pollution ([Katoto et al. 2019](#_ENREF_71)) ([Gascon et al. 2017](#_ENREF_52)). |
|  | **Water**  (e.g. exposure to blue space) | Mental health ([Gascon et al. 2017](#_ENREF_52)) |  |
|  | **Climate**  (e.g. microclimate, urban heat island) | All-cause mortality and cardiorespiratory morbidity ([Schinasi, Benmarhnia, and De Roos 2018](#_ENREF_112)). | Climate change ([Schinasi, Benmarhnia, and De Roos 2018](#_ENREF_112)) |

*Legend: CVD (Cardiovascular Disease), QoL (quality of life), SDGs (Sustainability Development Goals), Urban Heat Islands (UHI).*

# References

Abeykoon, A. H., R. Engler-Stringer, and N. Muhajarine. 2017. 'Health-related outcomes of new grocery store interventions: A systematic review', *Public Health Nutrition*, 20: 2236-48.

Aidala, A. A., M. G. Wilson, V. Shubert, D. Gogolishvili, J. Globerman, S. Rueda, A. K. Bozack, M. Caban, and S. B. Rourke. 2016. 'Housing status, medical care, and health outcomes among people living with HIV/AIDS: A systematic review', *American Journal of Public Health*, 106: e1-e23.

Ajrouche, R., G. Ielsch, E. Cléro, C. Roudier, D. Gay, J. Guillevic, D. Laurier, and A. Le Tertre. 2017. 'Quantitative Health Risk Assessment of Indoor Radon: A Systematic Review', *Radiation protection dosimetry*, 177: 69-77.

Akaraci, S., X. Feng, T. Suesse, B. Jalaludin, and T. Astell-Burt. 2020. 'A systematic review and meta-analysis of associations between green and blue spaces and birth outcomes', *International Journal of Environmental Research and Public Health*, 17.

Alaazi, D. A., and G. A. M. Aganah. 2020. 'Understanding the slum–health conundrum in sub-Saharan Africa: a proposal for a rights-based approach to health promotion in slums', *Global Health Promotion*, 27: 65-72.

Aletta, F., T. Oberman, and J. Kang. 2018. 'Associations between positive health-related effects and soundscapes perceptual constructs: A systematic review', *International Journal of Environmental Research and Public Health*, 15.

Algren, M. H., C. K. Bak, G. Berg-Beckhoff, and P. T. Andersen. 2015. 'Health-risk behaviour in deprived neighbourhoods compared with non-deprived neighbourhoods: A systematic literature review of quantitative observational studies', *PLoS ONE*, 10.

Allen, J. G., P. MacNaughton, J. G. C. Laurent, S. S. Flanigan, E. S. Eitland, and J. D. Spengler. 2015. 'Green Buildings and Health', *Current environmental health reports*, 2: 250-58.

Aromataris, E, and Z Munn. 2020. "JBI Manual for Evidence Synthesis." In.: JBI.

Aubry, T., G. Bloch, V. Brcic, A. Saad, O. Magwood, T. Abdalla, Q. Alkhateeb, E. Xie, C. Mathew, T. Hannigan, C. Costello, K. Thavorn, V. Stergiopoulos, P. Tugwell, and K. Pottie. 2020. 'Effectiveness of permanent supportive housing and income assistance interventions for homeless individuals in high-income countries: a systematic review', *The Lancet Public Health*, 5: e342-e60.

Audate, P. P., M. A. Fernandez, G. Cloutier, and A. Lebel. 2019. 'Scoping review of the impacts of urban agriculture on the determinants of health', *BMC Public Health*, 19.

Audrey, S., and H. Batista-Ferrer. 2015. 'Healthy urban environments for children and young people: A systematic review of intervention studies', *Health and Place*, 36: 97-117.

Bai, Xuemei, Alyson Surveyer, Thomas Elmqvist, Franz W Gatzweiler, Burak Güneralp, Susan Parnell, Anne-Helene Prieur-Richard, Paul Shrivastava, Jose Gabriel Siri, and Mark Stafford-Smith. 2016. 'Defining and advancing a systems approach for sustainable cities', *Current Opinion in Environmental Sustainability*, 23: 69-78.

Bailey, J., E. Gerasopoulos, D. Rojas-Rueda, and T. Benmarhnia. 2019. 'Potential health and equity co-benefits related to the mitigation policies reducing air pollution from residential wood burning in Athens, Greece', *Journal of Environmental Science and Health - Part A Toxic/Hazardous Substances and Environmental Engineering*, 54: 1144-51.

Barnard, L. S., D. J. Wexler, D. DeWalt, and S. A. Berkowitz. 2015. 'Material Need Support Interventions for Diabetes Prevention and Control: a Systematic Review', *Current Diabetes Reports*, 15.

Barone-Adesi, F., J. E. Dent, D. Dajnak, S. Beevers, H. R. Anderson, F. J. Kelly, D. G. Cook, and P. H. Whincup. 2015. 'Long-term exposure to primary traffic pollutants and lung function in children: Cross-sectional study and meta-analysis', *PLoS ONE*, 10.

Barros, P., L. Ng Fat, L. M. T. Garcia, A. D. Slovic, N. Thomopoulos, T. H. de Sá, P. Morais, and J. S. Mindell. 2019. 'Social consequences and mental health outcomes of living in high-rise residential buildings and the influence of planning, urban design and architectural decisions: A systematic review', *Cities*, 93: 263-72.

Beaudeau, P. 2018. 'A systematic review of the time series studies addressing the endemic risk of acute gastroenteritis according to drinkingwater operation conditions in urban areas of developed countries', *International Journal of Environmental Research and Public Health*, 15.

Bécares, L., M. E. Dewey, and J. Das-Munshi. 2018. 'Ethnic density effects for adult mental health: Systematic review and meta-analysis of international studies', *Psychological Medicine*, 48: 2054-72.

Benmarhnia, T., L. Rey, Y. Cartier, C. M. Clary, S. Deguen, and A. Brousselle. 2014. 'Addressing equity in interventions to reduce air pollution in urban areas: a systematic review', *International Journal of Public Health*, 59: 933-44.

Berghauser Pont, M. Y., P. G. Perg, P. A. Haupt, and A. Heyman. 2020. "A systematic review of the scientifically demonstrated effects of densification." In, edited by H. Wallbaum, A. Hollberg, L. Thuvander, P. Femenias, I. Kurkowska, K. Mjornell and C. Fudge. IOP Publishing Ltd.

Bhavsar, N. A., M. Kumar, and L. Richman. 2020. 'Defining gentrification for epidemiologic research: A systematic review', *PLoS ONE*, 15.

Bird, E. L., J. O. Ige, P. Pilkington, A. Pinto, C. Petrokofsky, and J. Burgess-Allen. 2018. 'Built and natural environment planning principles for promoting health: An umbrella review', *BMC Public Health*, 18.

Boothe, V. L., T. K. Boehmer, A. M. Wendel, and F. Y. Yip. 2014. 'Residential traffic exposure and childhood leukemia: A systematic review and meta-analysis', *American Journal of Preventive Medicine*, 46: 413-22.

Browning, M., and K. Lee. 2017. 'Within what distance does “greenness” best predict physical health? A systematic review of articles with gis buffer analyses across the lifespan', *International Journal of Environmental Research and Public Health*, 14.

Bulkeley, Harriet, and Michele Betsill. 2005. 'Rethinking Sustainable Cities: Multilevel Governance and the 'Urban' Politics of Climate Change', *Environmental Politics*, 14: 42-63.

Bulkeley, Harriet, and Kristine Kern. 2006. 'Local Government and the Governing of Climate Change in Germany and the UK', *Urban Studies*, 43: 2237-59.

Calogiuri, G., and S. Chroni. 2014. 'The impact of the natural environment on the promotion of active living: An integrative systematic review', *BMC Public Health*, 14.

Carlin, A., C. Perchoux, A. Puggina, K. Aleksovska, C. Buck, C. Burns, G. Cardon, S. Chantal, D. Ciarapica, G. Condello, T. Coppinger, C. Cortis, S. D’Haese, M. De Craemer, A. Di Blasio, S. Hansen, L. Iacoviello, J. Issartel, P. Izzicupo, L. Jaeschke, M. Kanning, A. Kennedy, J. Lakerveld, F. C. M. Ling, A. Luzak, G. Napolitano, J. A. Nazare, T. Pischon, A. Polito, A. Sannella, H. Schulz, R. Sohun, A. Steinbrecher, W. Schlicht, W. Ricciardi, C. Macdonncha, L. Capranica, and S. Boccia. 2017. 'A life course examination of the physical environmental determinants of physical activity behaviour: A “Determinants of Diet and Physical Activity” (DEDIPAC) umbrella systematic literature review', *PLoS ONE*, 12.

Carmona, M. 2019. 'Place value: place quality and its impact on health, social, economic and environmental outcomes', *Journal of Urban Design*, 24: 1-48.

Carnemolla, P., and C. Bridge. 2020. 'A scoping review of home modification interventions – Mapping the evidence base', *Indoor and Built Environment*, 29: 299-310.

Carrere, J., A. Reyes, L. Oliveras, A. Fernández, A. Peralta, A. M. Novoa, K. Pérez, and C. Borrell. 2020. 'The effects of cohousing model on people's health and wellbeing: A scoping review', *Public Health Reviews*, 41.

Chandrabose, M., J. N. Rachele, L. Gunn, A. Kavanagh, N. Owen, G. Turrell, B. Giles-Corti, and T. Sugiyama. 2019. 'Built environment and cardio-metabolic health: systematic review and meta-analysis of longitudinal studies', *Obesity Reviews*, 20: 41-54.

Chaparro, R., S. Melendi, M. Santero, M. Seijo, N. Elorriaga, M. Belizan, A. Rubinstein, and V. Irazola. 2020. 'A review of assessment indicators used by Healthy Municipalities and Communities Program in Latin America and the Caribbean region', *Health Promotion International*, 35: 714-29.

Chastin, S. F. M., C. Buck, E. Freiberger, M. Murphy, J. Brug, G. Cardon, G. O'Donoghue, I. Pigeot, J. M. Oppert, and Dedipac consortium on behalf of the. 2015. 'Systematic literature review of determinants of sedentary behaviour in older adults: A DEDIPAC study', *International Journal of Behavioral Nutrition and Physical Activity*, 12.

Cho, H. Y., M. MacLachlan, M. Clarke, and H. Mannan. 2016. 'Accessible home environments for people with functional limitations: A systematic review', *International Journal of Environmental Research and Public Health*, 13.

Crane, Melanie, Simon Lloyd, Andy Haines, Ding Ding, Emma Hutchinson, Kristine Belesova, Michael Davies, David Osrin, Nici Zimmermann, Anthony Capon, Paul Wilkinson, and Catalina Turcu. 2021. 'Transforming cities for sustainability: A health perspective', *Environment International*, 147: 106366.

Cyril, S., J. C. Oldroyd, and A. Renzaho. 2013. 'Urbanisation, urbanicity, and health: A systematic review of the reliability and validity of urbanicity scales', *BMC Public Health*, 13.

Davey, Brooke, Raina Sinha, Ji Hyun Lee, Marissa Gauthier, and Glenn Flores. 2020. 'Social determinants of health and outcomes for children and adults with congenital heart disease: a systematic review', *Pediatric research*.

de Keijzer, C., M. Bauwelinck, and P. Dadvand. 2020. 'Long-Term Exposure to Residential Greenspace and Healthy Ageing: a Systematic Review', *Current environmental health reports*, 7: 65-88.

de Keijzer, C., M. Gascon, M. J. Nieuwenhuijsen, and P. Dadvand. 2016. 'Long-Term Green Space Exposure and Cognition Across the Life Course: a Systematic Review', *Current environmental health reports*, 3: 468-77.

Decker, M. J., S. Isquick, L. Tilley, Q. Zhi, A. Gutman, W. Luong, and C. D. Brindis. 2018. 'Neighborhoods matter. A systematic review of neighborhood characteristics and adolescent reproductive health outcomes', *Health and Place*, 54: 178-90.

Doocy, S., A. Daniels, C. Packer, A. Dick, and T. D. Kirsch. 2013. 'The Human Impact of Earthquakes: A Historical Review of Events 1980-2009 and Systematic Literature Review', *PLoS Currents*.

Downing, J. 2016. 'The health effects of the foreclosure crisis and unaffordable housing: A systematic review and explanation of evidence', *Social Science and Medicine*, 162: 88-96.

Durand, C. P., M. Andalib, G. F. Dunton, J. Wolch, and M. A. Pentz. 2011. 'A systematic review of built environment factors related to physical activity and obesity risk: Implications for smart growth urban planning', *Obesity Reviews*, 12: e173-e82.

Dzhambov, A. M., and D. D. Dimitrova. 2018. 'Residential road traffic noise as a risk factor for hypertension in adults: Systematic review and meta-analysis of analytic studies published in the period 2011–2017', *Environmental Pollution*, 240: 306-18.

Eisenberg, Y., K. A. Vanderbom, and V. Vasudevan. 2017. 'Does the built environment moderate the relationship between having a disability and lower levels of physical activity? A systematic review', *Preventive Medicine*, 95: S75-S84.

Felappi, J. F., J. H. Sommer, T. Falkenberg, W. Terlau, and T. Kötter. 2020. 'Green infrastructure through the lens of “One Health”: A systematic review and integrative framework uncovering synergies and trade-offs between mental health and wildlife support in cities', *Science of the Total Environment*, 748.

Fenwick, E., C. Macdonald, and H. Thomson. 2013. 'Economic analysis of the health impacts of housing improvement studies: A systematic review', *Journal of Epidemiology and Community Health*, 67: 835-45.

Garin, N., B. Olaya, M. Miret, J. L. Ayuso-Mateos, M. Power, P. Bucciarelli, and J. M. Haro. 2014. 'Built environment and elderly population health: A comprehensive literature review', *Clinical Practice and Epidemiology in Mental Health*, 10: 103-15.

Gascon, M., M. T. Mas, D. Martínez, P. Dadvand, J. Forns, A. Plasència, and M. J. Nieuwenhuijsen. 2015. 'Mental health benefits of long-term exposure to residential green and blue spaces: A systematic review', *International Journal of Environmental Research and Public Health*, 12: 4354-79.

Gascon, M., W. Zijlema, C. Vert, M. P. White, and M. J. Nieuwenhuijsen. 2017. 'Outdoor blue spaces, human health and well-being: A systematic review of quantitative studies', *International Journal of Hygiene and Environmental Health*, 220: 1207-21.

Giles-Corti, Billie, Melanie Lowe, and Jonathan Arundel. 2019. 'Achieving the SDGs: Evaluating indicators to be used to benchmark and monitor progress towards creating healthy and sustainable cities', *Health Policy*.

Gong, Y., S. Palmer, J. Gallacher, T. Marsden, and D. Fone. 2016. 'A systematic review of the relationship between objective measurements of the urban environment and psychological distress', *Environment International*, 96: 48-57.

Habash, M., P. Gogna, D. Krewski, and R. Habash. 2019. 'Scoping review of the potential health effects of exposure to extremely low-frequency electric and magnetic fields', *Critical Reviews in Biomedical Engineering*, 47: 323-47.

Hamra, G. B., F. Laden, A. J. Cohen, O. Raaschou-Nielsen, M. Brauer, and D. Loomis. 2015. 'Lung cancer and exposure to nitrogen dioxide and traffic: A systematic review and meta-analysis', *Environmental Health Perspectives*, 123: 1107-12.

Hartley, K., P. Ryan, C. Brokamp, and G. L. Gillespie. 2020. 'Effect of greenness on asthma in children: A systematic review', *Public Health Nursing*, 37: 453-60.

Hawkes, Sarah, Bhupinder K Aulakh, Nidhee Jadeja, Michelle Jimenez, Kent Buse, Iqbal Anwar, Sandhya Barge, M Oladoyin Odubanjo, Abhay Shukla, and Abdul Ghaffar. 2016. 'Strengthening capacity to apply health research evidence in policy making: experience from four countries', *Health policy and planning*, 31: 161-70.

Heijnen, M., O. Cumming, R. Peletz, G. K. S. Chan, J. Brown, K. Baker, and T. Clasen. 2014. 'Shared sanitation versus individual household latrines: A systematic review of health outcomes', *PLoS ONE*, 9.

Henson, R. M., A. Ortigoza, K. Martinez-Folgar, F. Baeza, W. Caiaffa, A. Vives Vergara, A. V. Diez Roux, and G. Lovasi. 2020. 'Evaluating the health effects of place-based slum upgrading physical environment interventions: A systematic review (2012–2018)', *Social Science and Medicine*, 261.

Higgins, Julian PT, James Thomas, Jacqueline Chandler, Miranda Cumpston, Tianjing Li, Matthew J Page, and Vivian A Welch. 2019. *Cochrane handbook for systematic reviews of interventions* (John Wiley & Sons).

Houghton, A., and C. Castillo-Salgado. 2017. 'Health co-benefits of green building design strategies and community resilience to urban flooding: A systematic review of the evidence', *International Journal of Environmental Research and Public Health*, 14.

———. 2019. 'Associations between green building design strategies and community health resilience to extreme heat events: A systematic review of the evidence', *International Journal of Environmental Research and Public Health*, 16.

Iacovou, M., D. C. Pattieson, H. Truby, and C. Palermo. 2013. 'Social health and nutrition impacts of community kitchens: A systematic review', *Public Health Nutrition*, 16: 535-43.

Ige, J., P. Pilkington, J. Orme, B. Williams, E. Prestwood, D. Black, L. Carmichael, and G. Scally. 2019. 'The relationship between buildings and health: A systematic review', *Journal of Public Health (United Kingdom)*, 41: E121-E32.

Ishaq, S., R. Sadiq, S. Farooq, G. Chhipi-Shrestha, and K. Hewage. 2020. 'Investigating the public health risks of low impact developments at residential, neighbourhood, and municipal levels', *Science of the Total Environment*, 744.

Islam, M. Z., J. Johnston, and P. D. Sly. 2020. 'Green space and early childhood development: A systematic review', *Reviews on Environmental Health*, 35: 189-200.

Jevons, R., C. Carmichael, A. Crossley, and A. Bone. 2016. 'Minimum indoor temperature threshold recommendations for English homes in winter – A systematic review', *Public Health*, 136: 4-12.

Jung, Y. T., R. J. Hum, W. Lou, and Y. L. Cheng. 2017. 'Effects of neighbourhood and household sanitation conditions on diarrhea morbidity: Systematic review and meta-analysis', *PLoS ONE*, 12.

Kabisch, N., M. van den Bosch, and R. Lafortezza. 2017. 'The health benefits of nature-based solutions to urbanization challenges for children and the elderly – A systematic review', *Environmental Research*, 159: 362-73.

Katoto, P. D. M. C., L. Byamungu, A. S. Brand, J. Mokaya, H. Strijdom, N. Goswami, P. De Boever, T. S. Nawrot, and B. Nemery. 2019. 'Ambient air pollution and health in Sub-Saharan Africa: Current evidence, perspectives and a call to action', *Environmental Research*, 173: 174-88.

Khosravipour, M., and P. Khanlari. 2020. 'The association between road traffic noise and myocardial infarction: A systematic review and meta-analysis', *Science of the Total Environment*, 731.

Kondo, M. C., J. M. Fluehr, T. McKeon, and C. C. Branas. 2018. 'Urban green space and its impact on human health', *International Journal of Environmental Research and Public Health*, 15.

Kondo, M. C., S. F. Jacoby, and E. C. South. 2018. 'Does spending time outdoors reduce stress? A review of real-time stress response to outdoor environments', *Health and Place*, 51: 136-50.

Lai, H., E. J. Flies, P. Weinstein, and A. Woodward. 2019. 'The impact of green space and biodiversity on health', *Frontiers in Ecology and the Environment*, 17: 383-90.

Leal, C., and B. Chaix. 2011. 'The influence of geographic life environments on cardiometabolic risk factors: A systematic review, a methodological assessment and a research agenda', *Obesity Reviews*, 12: 217-30.

Lee, C., G. Burgess, I. Kuhn, A. Cowan, and L. Lafortune. 2020. 'Community exchange and time currencies: a systematic and in-depth thematic review of impact on public health outcomes', *Public Health*, 180: 117-28.

Lee, K. J., H. Moon, H. R. Yun, E. L. Park, A. R. Park, H. Choi, K. Hong, and J. Lee. 2020. 'Greenness, civil environment, and pregnancy outcomes: Perspectives with a systematic review and meta-analysis', *Environmental Health: A Global Access Science Source*, 19.

Lee, K. K., R. Bing, J. Kiang, S. Bashir, N. Spath, D. Stelzle, K. Mortimer, A. Bularga, D. Doudesis, S. S. Joshi, F. Strachan, S. Gumy, H. Adair-Rohani, E. F. Attia, M. H. Chung, M. R. Miller, D. E. Newby, N. L. Mills, D. A. McAllister, and A. S. V. Shah. 2020. 'Adverse health effects associated with household air pollution: a systematic review, meta-analysis, and burden estimation study', *The Lancet Global Health*, 8: e1427-e34.

Levy-Storms, L., L. Chen, and A. Loukaitou-Sideris. 2018. 'Older Adults' needs and preferences for open space and physical activity in and near parks: A systematic review', *Journal of Aging and Physical Activity*, 26: 682-96.

Lorenc, T., M. Petticrew, M. Whitehead, D. Neary, S. Clayton, K. Wright, H. Thomson, S. Cummins, A. Sowden, and A. Renton. 2013. 'Environmental interventions to reduce fear of crime: systematic review of effectiveness', *Systematic reviews*, 2: 30.

Ma, X., I. Longley, J. Gao, and J. Salmond. 2020. 'Assessing schoolchildren's exposure to air pollution during the daily commute - A systematic review', *Science of the Total Environment*, 737.

Macmillan, F., E. S. George, X. Feng, D. Merom, A. Bennie, A. Cook, T. Sanders, G. Dwyer, B. Pang, J. M. Guagliano, G. S. Kolt, and T. Astell-Burt. 2018. 'Do natural experiments of changes in neighborhood built environment impact physical activity and diet? A systematic review', *International Journal of Environmental Research and Public Health*, 15.

Malambo, P., A. P. Kengne, A. De Villiers, E. V. Lambert, and T. Puoane. 2016. 'Built environment, selected risk factors and major cardiovascular disease outcomes: A systematic review', *PLoS ONE*, 11.

Marmot, Michael. 2005. 'Social determinants of health inequalities', *The lancet*, 365: 1099-104.

McCormick, R. 2017. 'Does Access to Green Space Impact the Mental Well-being of Children: A Systematic Review', *Journal of Pediatric Nursing*, 37: 3-7.

McCrorie, P. R. W., C. Fenton, and A. Ellaway. 2014. 'Combining GPS, GIS, and accelerometry to explore the physical activity and environment relationship in children and young people - a review', *International Journal of Behavioral Nutrition and Physical Activity*, 11.

Möller, H., F. Haigh, R. Hayek, and L. Veerman. 2020. 'What is the best practice method for quantifying the health and economic benefits of active transport?', *International Journal of Environmental Research and Public Health*, 17: 1-16.

Moore, T. H. M., J. M. Kesten, J. A. López-López, S. Ijaz, A. McAleenan, A. Richards, S. Gray, J. Savović, and S. Audrey. 2018. 'The effects of changes to the built environment on the mental health and well-being of adults: Systematic review', *Health and Place*, 53: 237-57.

Moran, M., J. Van Cauwenberg, R. Hercky-Linnewiel, E. Cerin, B. Deforche, and P. Plaut. 2014. 'Understanding the relationships between the physical environment and physical activity in older adults: A systematic review of qualitative studies', *International Journal of Behavioral Nutrition and Physical Activity*, 11.

Naldzhiev, D., D. Mumovic, and M. Strlic. 2020. 'Polyurethane insulation and household products – A systematic review of their impact on indoor environmental quality', *Building and Environment*, 169.

Nordbø, E. C. A., H. Nordh, R. K. Raanaas, and G. Aamodt. 2018. 'GIS-derived measures of the built environment determinants of mental health and activity participation in childhood and adolescence: A systematic review', *Landscape and Urban Planning*, 177: 19-37.

Núñez-González, S., J. A. Delgado-Ron, C. Gault, A. Lara-Vinueza, D. Calle-Celi, R. Porreca, and D. Simancas-Racines. 2020. 'Overview of "systematic Reviews" of the Built Environment's Effects on Mental Health', *Journal of Environmental and Public Health*, 2020.

Nussbaumer-Streit, B., V. Mayr, A. I. Dobrescu, G. Wagner, A. Chapman, L. M. Pfadenhauer, S. Lohner, S. K. Lhachimi, L. K. Busert, and G. Gartlehner. 2020. 'Household interventions for secondary prevention of domestic lead exposure in children', *Cochrane Database of Systematic Reviews*, 2020.

Parker, J., and M. E. Z. de Baro. 2019. 'Green infrastructure in the urban environment: A systematic quantitative review', *Sustainability (Switzerland)*, 11.

Patterson, R., E. Webb, T. Hone, C. Millett, and A. A. Laverty. 2019. 'Associations of Public Transportation Use with Cardiometabolic Health: A Systematic Review and Meta-Analysis', *American Journal of Epidemiology*, 188: 785-95.

Pega, F., and N. Wilson. 2016. 'A systematic review of health economic analyses of housing improvement interventions and insecticide-treated bednets in the home', *PLoS ONE*, 11.

Pérez, E., C. Braën, G. Boyer, G. Mercille, É Rehany, V. Deslauriers, A. Bilodeau, and L. Potvin. 2020. 'Neighbourhood community life and health: A systematic review of reviews', *Health and Place*, 61.

Peris, E., and B. Fenech. 2020. 'Associations and effect modification between transportation noise, self-reported response to noise and the wider determinants of health: A narrative synthesis of the literature', *Science of the Total Environment*, 748.

Quansah, R., S. Semple, C. A. Ochieng, S. Juvekar, F. A. Armah, I. Luginaah, and J. Emina. 2017. 'Effectiveness of interventions to reduce household air pollution and/or improve health in homes using solid fuel in low-and-middle income countries: A systematic review and meta-analysis', *Environment International*, 103: 73-90.

Ramaswami, Anu, Armistead G Russell, Patricia J Culligan, Karnamadakala Rahul Sharma, and Emani Kumar. 2016. 'Meta-principles for developing smart, sustainable, and healthy cities', *Science*, 352: 940-43.

Rhodes, R. E., B. E. Saelens, and C. Sauvage-Mar. 2018. 'Understanding Physical Activity through Interactions Between the Built Environment and Social Cognition: A Systematic Review', *Sports Medicine*, 48: 1893-912.

Rocha, N. P., A. Dias, G. Santinha, M. Rodrigues, A. Queirós, and C. Rodrigues. 2019. "Smart Cities and Public Health: A Systematic Review." In, edited by M. M. Cruz-Cunha, J. E. Varajao, R. Martinho, R. Rijo, E. Peres and D. Domingos, 516-23. Elsevier B.V.

Rojas-Rueda, D., M. J. Nieuwenhuijsen, M. Gascon, D. Perez-Leon, and P. Mudu. 2019. 'Green spaces and mortality: a systematic review and meta-analysis of cohort studies', *The Lancet Planetary Health*, 3: e469-e77.

Rosso, A. L., A. H. Auchincloss, and Y. L. Michael. 2011. 'The urban built environment and mobility in older adults: A comprehensive review', *Journal of Aging Research*, 2011.

Rugel, E. J., and M. Brauer. 2020. 'Quiet, clean, green, and active: A Navigation Guide systematic review of the impacts of spatially correlated urban exposures on a range of physical health outcomes', *Environmental Research*, 185.

Rydin, Yvonne, Ana Bleahu, Michael Davies, Julio D Dávila, Sharon Friel, Giovanni De Grandis, Nora Groce, Pedro C Hallal, Ian Hamilton, and Philippa Howden-Chapman. 2012. 'Shaping cities for health: complexity and the planning of urban environments in the 21st century', *The Lancet*, 379: 2079-108.

Saitta, M., H. Devan, P. Boland, and M. A. Perry. 2019. 'Park-based physical activity interventions for persons with disabilities: A mixed-methods systematic review', *Disability and Health Journal*, 12: 11-23.

Salgado, M., J. Madureira, A. S. Mendes, A. Torres, J. P. Teixeira, and M. D. Oliveira. 2020. 'Environmental determinants of population health in urban settings. A systematic review', *BMC Public Health*, 20.

Sallis, James F, Fiona Bull, Ricky Burdett, Lawrence D Frank, Peter Griffiths, Billie Giles-Corti, and Mark Stevenson. 2016. 'Use of science to guide city planning policy and practice: how to achieve healthy and sustainable future cities', *The lancet*, 388: 2936-47.

Sánchez-González, D., F. Rojo-Pérez, V. Rodríguez-Rodríguez, and G. Fernández-Mayoralas. 2020. 'Environmental and psychosocial interventions in age-friendly communities and active ageing: A systematic review', *International Journal of Environmental Research and Public Health*, 17: 1-35.

Schinasi, L. H., T. Benmarhnia, and A. J. De Roos. 2018. 'Modification of the association between high ambient temperature and health by urban microclimate indicators: A systematic review and meta-analysis', *Environmental Research*, 161: 168-80.

Schneidera, Carmen Huckel, and Fiona Blythb. 2017. 'Challenges of integrating evidence into health policy and planning: linking multiple disciplinary approaches', *work*, 1: 5-7.

Schüle, S. A., and G. Bolte. 2015. 'Interactive and independent associations between the socioeconomic and objective built environment on the neighbourhood level and individual health: A systematic review of multilevel studies', *PLoS ONE*, 10.

Schüle, S. A., L. K. Hilz, S. Dreger, and G. Bolte. 2019. 'Social inequalities in environmental resources of green and blue spaces: A review of evidence in the WHO European region', *International Journal of Environmental Research and Public Health*, 16.

Seligman, Martin EP. 2004. *Authentic happiness: Using the new positive psychology to realize your potential for lasting fulfillment* (Simon and Schuster).

Seligman, ME. 2011. 'Flourish: a visionary new understanding of happiness and well-being', *Policy*, 27: 60-1.

Senthilkumaran, M., G. Nazari, J. C. MacDermid, K. Roche, and K. Sopko. 2019. 'Effectiveness of home fire safety interventions. A systematic review and metaanalysis', *PLoS ONE*, 14.

Sharpe, R. A., N. Bearman, C. R. Thornton, K. Husk, and N. J. Osborne. 2015. 'Indoor fungal diversity and asthma: A meta-analysis and systematic review of risk factors', *Journal of Allergy and Clinical Immunology*, 135: 110-22.

Shuvo, F. K., X. Feng, S. Akaraci, and T. Astell-Burt. 2020. 'Urban green space and health in low and middle-income countries: A critical review', *Urban Forestry and Urban Greening*, 52.

Singh, A., L. Daniel, E. Baker, and R. Bentley. 2019. 'Housing Disadvantage and Poor Mental Health: A Systematic Review', *American Journal of Preventive Medicine*, 57: 262-72.

Smelser, Neil J, and Paul B Baltes. 2001. *International encyclopedia of the social & behavioral sciences* (Elsevier Amsterdam).

Stankov, I., L. M. T. Garcia, M. A. Mascolli, F. Montes, J. D. Meisel, N. Gouveia, O. L. Sarmiento, D. A. Rodriguez, R. A. Hammond, W. T. Caiaffa, and A. V. Diez Roux. 2020. 'A systematic review of empirical and simulation studies evaluating the health impact of transportation interventions', *Environmental Research*, 186.

Stewart, G., N. K. Anokye, and S. Pokhrel. 2015. 'What interventions increase commuter cycling? A systematic review', *BMJ Open*, 5.

Tagiyeva, N., and A. Sheikh. 2014. 'Domestic exposure to volatile organic compounds in relation to asthma and allergy in children and adults', *Expert Review of Clinical Immunology*, 10: 1611-39.

Tanner, L. M., S. Moffatt, E. M. G. Milne, S. D. H. Mills, and M. White. 2013. 'Socioeconomic and behavioural risk factors for adverse winter health and social outcomes in economically developed countries: A systematic review of quantitative observational studies', *Journal of Epidemiology and Community Health*, 67: 1061-67.

Thomson, H., and S. Thomas. 2015. 'Developing empirically supported theories of change for housing investment and health', *Social Science and Medicine*, 124: 205-14.

Tsai, A. C. 2015. 'Home foreclosure, health, and mental health: A systematic review of individual, aggregate, and contextual associations', *PLoS ONE*, 10.

Tseng, E., A. Zhang, O. Shogbesan, K. A. Gudzune, R. F. Wilson, H. Kharrazi, L. J. Cheskin, E. B. Bass, and W. L. Bennett. 2018. 'Effectiveness of Policies and Programs to Combat Adult Obesity: a Systematic Review', *Journal of General Internal Medicine*, 33: 1990-2001.

Turcu, C, L Andres, M Crane, and M Ding. 2021. 'The value of interdisciplinarity: a critical reflection on urban sustainability research', *Buildings & Cities*.

Turley, R., R. Saith, N. Bhan, E. Rehfuess, and B. Carter. 2013. 'Slum upgrading strategies involving physical environment and infrastructure interventions and their effects on health and socio-economic outcomes', *Cochrane Database of Systematic Reviews*, 2013.

Twohig-Bennett, C., and A. Jones. 2018. 'The health benefits of the great outdoors: A systematic review and meta-analysis of greenspace exposure and health outcomes', *Environmental Research*, 166: 628-37.

UN. 2018. '2018 Revision of World Urbanization Prospects', *May*, 16: 2018.

Van Boven, F. E., N. W. De Jong, G. J. Braunstahl, L. R. Arends, and R. Gerth Van Wijk. 2020. 'Effectiveness of the Air Purification Strategies for the Treatment of Allergic Asthma: A Meta-Analysis', *International Archives of Allergy and Immunology*, 181: 395-402.

van den Berg, M., W. Wendel-Vos, M. van Poppel, H. Kemper, W. van Mechelen, and J. Maas. 2015. 'Health benefits of green spaces in the living environment: A systematic review of epidemiological studies', *Urban Forestry and Urban Greening*, 14: 806-16.

Van Kempen, E., and W. Babisch. 2012. 'The quantitative relationship between road traffic noise and hypertension: A meta-analysis', *Journal of Hypertension*, 30: 1075-86.

Vanaken, G. J., and M. Danckaerts. 2018. 'Impact of green space exposure on children’s and adolescents’ mental health: A systematic review', *International Journal of Environmental Research and Public Health*, 15.

Vardoulakis, S., E. Giagloglou, S. Steinle, A. Davis, A. Sleeuwenhoek, K. S. Galea, K. Dixon, and J. O. Crawford. 2020. 'Indoor exposure to selected air pollutants in the home environment: A systematic review', *International Journal of Environmental Research and Public Health*, 17: 1-24.

Vásquez-Vera, H., L. Palència, I. Magna, C. Mena, J. Neira, and C. Borrell. 2017. 'The threat of home eviction and its effects on health through the equity lens: A systematic review', *Social Science and Medicine*, 175: 199-208.

Venkataramanan, V., A. I. Packman, D. R. Peters, D. Lopez, D. J. McCuskey, R. I. McDonald, W. M. Miller, and S. L. Young. 2019. 'A systematic review of the human health and social well-being outcomes of green infrastructure for stormwater and flood management', *Journal of Environmental Management*, 246: 868-80.

Vyncke, V., B. De Clercq, V. Stevens, C. Costongs, G. Barbareschi, S. H. Jónsson, S. D. Curvo, V. Kebza, C. Currie, and L. Maes. 2013. 'Does neighbourhood social capital aid in levelling the social gradient in the health and well-being of children and adolescents? A literature review', *BMC Public Health*, 13.

Whitmee, S., A. Haines, C. Beyrer, F. Boltz, A. G. Capon, B. F. De Souza Dias, A. Ezeh, H. Frumkin, P. Gong, P. Head, R. Horton, G. M. Mace, R. Marten, S. S. Myers, S. Nishtar, S. A. Osofsky, S. K. Pattanayak, M. J. Pongsiri, C. Romanelli, A. Soucat, J. Vega, and D. Yach. 2015. 'Safeguarding human health in the Anthropocene epoch: Report of the Rockefeller Foundation-Lancet Commission on planetary health', *The Lancet*, 386: 1973-2028.

WHO. 2018. "WHO housing and health guidelines." In. Geneve: World Health Organization (WHO).

———. 2020. "Integrating health in urban and territorial planning: a sourcebook." In. Geneve: World Health Organization.

Yuan, Yin, Feng Huang, Fan Lin, Pengyi Zhu, and Pengli Zhu. 2020. 'Green space exposure on mortality and cardiovascular outcomes in older adults: a systematic review and meta-analysis of observational studies', *Aging clinical and experimental research*.

Zhang, G., D. V. Poulsen, V. L. Lygum, S. S. Corazon, M. C. Gramkow, and U. K. Stigsdotter. 2017. 'Health-promoting nature access for people with mobility impairments: A systematic review', *International Journal of Environmental Research and Public Health*, 14.
