# Supplementary material for: A multi-scalar perspective on health and urban housing: an umbrella review
Source: Build Cities. Author manuscript; Available in PMC 2021 Nov 3. (PMC7611930; doi:10.5334/bc.119)
Supplement: Supplementary Table [file EMS137021-supplement-Table_S3.docx]

Table 3 – Systematic reviews reporting on URBAN SYSTEM determiants of health

| *Determinant* | *Specific aspects* | *Reported human health impact or outcomes* | *Reported planetary health impacts or outcomes* | *References* |
| --- | --- | --- | --- | --- |
| **Spatial Planning** | **Informality**  (e.g. slums etc) | Slum infrastructure and material conditions AND physical and mental health effects. | - | (Alaazi and Aganah 2020) |
|  |  | Slum upgrade strategies AND physical health (including maternal health), as well as QoL and social capital. | - | (Turley et al. 2013) |
|  |  | Slum upgrade strategies AND physical and mental health, as well as QoL and social capital. | Rapid urbanisation, effects on the environment, and the SDGs. | (Henson et al. 2020) |
|  | **Urban infrastructure**  (e.g. water, urban structure, power lines etc) | Drinking water AND gastroenteritis. | - | (Beaudeau 2018) |
|  |  | Structural soundness of city AND mortality, morbidity, and displacement in the event of an earthquake. | - | (Doocy et al. 2013) |
|  |  | Exposure to overhead power lines AND cancer, CVD, reproductive outcomes and neurodegenerative disease. | - | (Habash et al. 2019) |
|  | **Type of development**  (e.g. densification, buit form typology, urbanicity) | Urban density AND walkability, obesity and mental health. | Densification benefits for achieving SDGs | (Berghauser Pont et al. 2020) |
|  |  | Built attributes influencing PA AND cardio-metabolic diseases. |  | (Chandrabose et al. 2019) |
|  |  | Urbanicity AND health behaviours (PA, F&V consumption) and biomarkers (BMI). |  | (Chandrabose et al. 2019) |
|  | **Masterplanning**  (e.g. urban designing for: active ageing, healthcare access;, smart growth, smart city; landscaping etc) | Planning for active aging AND fall risk of reduction as well as promotion of social interaction. | - | (Sánchez-González et al. 2020) |
|  |  | Built (including health care access) and natural urban environment AND mortality and morbidity. | Air pollution, noise, soil, water quality | (Salgado et al. 2020) |
|  |  | Urban planning including block design, housing quality, land use mix, and connectivity AND mental health. | - | (Gong et al. 2016) |
|  |  | Urban green and blue space AND the health of children and the elderly. | Urban noise, UHI, loss of green/blue space | (Kabisch, van den Bosch, and Lafortezza 2017) |
|  |  | Built environment (including density, land use mix, connectivity and green space) AND PA, mental health and QoL. | - | (Nordbø et al. 2018) |
|  |  | Urban planning tracked via GPS, GIS and accelerometry AND physical activity. | - | (McCrorie, Fenton, and Ellaway 2014) |
|  |  | "Smart Growth" approach to urban planning (including housing diversity, walkability, and community collaboration) AND PA and BMI. | - | (Durand et al. 2011) |
|  |  | Smart city infrastructure (including surveillance and behaviour prompts) AND healthy lifestyle promotion and disease prevalence. | Environmental conditions | (Rocha et al. 2019) |
| **Housing system** | **Vulnerability**  (e.g. housing status, housing insecurity, housing instability, social housing, foreclosure, evictions, housing precarity etc) | Housing status AND medical care and health outcomes among people with HIV | - | (Aidala et al. 2016) |
|  |  | Housing insecurity AND physical and mental health plus health behaviours. | - | (Vásquez-Vera et al. 2017) |
|  |  | Social determinants of health (including housing instability) AND congenital heart disease | -  - | (Davey et al. 2020) |
|  |  | Permanent supportive social housing AND health and health-related social outcomes (e.g. employment and income). | - | (Aubry et al. 2020) |
|  |  | Experiencing a foreclosure and/or living near foreclosures AND mental health. | - | (Downing 2016) |
|  |  | Home foreclosure AND physical and mental health plus health behaviours. | - | (Tsai 2015) |
|  |  | Socioeconomic, housing or behavioural factors AND cold weather-related adverse health or social outcomes. | Energy access (fuel poverty) | (Tanner et al. 2013) |
|  |  | Housing disadvantage (based on tenure, precarity, and physical characteristics) AND mental health. | - | (Singh et al. 2019) |
|  | **Policy**  (e.g. housing material support, healthy municipality strategy etc) | Interventions providing material support (including housing and food) AND diabetes incidence. | - | (Singh et al. 2019) |
|  |  | Programs, policies, and built-environment changes AND adult obesity. |  | (Tseng et al. 2018) |
|  |  | Healthy Municipality Communities Strategies (including material and social interventions) AND community health status. | - | (Chaparro et al. 2020) |
| **Ecosystems** | **Air**  (e.g. pollution etc) | Interventions aiming to reduce air pollution AND mortality and equity effects. | Air pollution (PM, NO2) | (Benmarhnia et al. 2014) |
|  |  | Outdoor air pollution levels AND cardio-respiratory health. | Air pollution | (Katoto et al. 2019) |
|  | **Water**  (e.g. exposure to blue space etc) | Long-term exposure to residential green and blue spaces AND mental health. | - | (Gascon et al. 2017) |
|  | **Climate**  (e.g. microclimate, UHI etc) | Microclimate indicators that modify UHI effect AND all-cause mortality plus cardio- and respiratory- morbidity | Climate change | (Schinasi, Benmarhnia, and De Roos 2018) |

Legend: BMI (body mass index), CO2 (Carbon dioxide), CVD (Cardiovascular Disease), ED (Emergency department), F&V(fruit and vegetable), NO2 (Nitrogen Dioxide), PA (physical activity), PM (particulate matter), QoL (quality of life), SDG (Sustainability Development Goals), Urban Heat Islands (UHI)

Aidala, A. A., M. G. Wilson, V. Shubert, D. Gogolishvili, J. Globerman, S. Rueda, A. K. Bozack, M. Caban, and S. B. Rourke. 2016. 'Housing status, medical care, and health outcomes among people living with HIV/AIDS: A systematic review', *American Journal of Public Health*, 106: e1-e23.

Alaazi, D. A., and G. A. M. Aganah. 2020. 'Understanding the slum–health conundrum in sub-Saharan Africa: a proposal for a rights-based approach to health promotion in slums', *Global Health Promotion*, 27: 65-72.

Aubry, T., G. Bloch, V. Brcic, A. Saad, O. Magwood, T. Abdalla, Q. Alkhateeb, E. Xie, C. Mathew, T. Hannigan, C. Costello, K. Thavorn, V. Stergiopoulos, P. Tugwell, and K. Pottie. 2020. 'Effectiveness of permanent supportive housing and income assistance interventions for homeless individuals in high-income countries: a systematic review', *The Lancet Public Health*, 5: e342-e60.

Beaudeau, P. 2018. 'A systematic review of the time series studies addressing the endemic risk of acute gastroenteritis according to drinkingwater operation conditions in urban areas of developed countries', *International Journal of Environmental Research and Public Health*, 15.

Benmarhnia, T., L. Rey, Y. Cartier, C. M. Clary, S. Deguen, and A. Brousselle. 2014. 'Addressing equity in interventions to reduce air pollution in urban areas: a systematic review', *International Journal of Public Health*, 59: 933-44.

Berghauser Pont, M. Y., P. G. Perg, P. A. Haupt, and A. Heyman. 2020. "A systematic review of the scientifically demonstrated effects of densification." In, edited by H. Wallbaum, A. Hollberg, L. Thuvander, P. Femenias, I. Kurkowska, K. Mjornell and C. Fudge. IOP Publishing Ltd.

Chandrabose, M., J. N. Rachele, L. Gunn, A. Kavanagh, N. Owen, G. Turrell, B. Giles-Corti, and T. Sugiyama. 2019. 'Built environment and cardio-metabolic health: systematic review and meta-analysis of longitudinal studies', *Obesity Reviews*, 20: 41-54.

Chaparro, R., S. Melendi, M. Santero, M. Seijo, N. Elorriaga, M. Belizan, A. Rubinstein, and V. Irazola. 2020. 'A review of assessment indicators used by Healthy Municipalities and Communities Program in Latin America and the Caribbean region', *Health Promotion International*, 35: 714-29.

Davey, Brooke, Raina Sinha, Ji Hyun Lee, Marissa Gauthier, and Glenn Flores. 2020. 'Social determinants of health and outcomes for children and adults with congenital heart disease: a systematic review', *Pediatric research*.

Doocy, S., A. Daniels, C. Packer, A. Dick, and T. D. Kirsch. 2013. 'The Human Impact of Earthquakes: A Historical Review of Events 1980-2009 and Systematic Literature Review', *PLoS Currents*.

Downing, J. 2016. 'The health effects of the foreclosure crisis and unaffordable housing: A systematic review and explanation of evidence', *Social Science and Medicine*, 162: 88-96.

Durand, C. P., M. Andalib, G. F. Dunton, J. Wolch, and M. A. Pentz. 2011. 'A systematic review of built environment factors related to physical activity and obesity risk: Implications for smart growth urban planning', *Obesity Reviews*, 12: e173-e82.

Gascon, M., W. Zijlema, C. Vert, M. P. White, and M. J. Nieuwenhuijsen. 2017. 'Outdoor blue spaces, human health and well-being: A systematic review of quantitative studies', *International Journal of Hygiene and Environmental Health*, 220: 1207-21.

Gong, Y., S. Palmer, J. Gallacher, T. Marsden, and D. Fone. 2016. 'A systematic review of the relationship between objective measurements of the urban environment and psychological distress', *Environment International*, 96: 48-57.

Habash, M., P. Gogna, D. Krewski, and R. Habash. 2019. 'Scoping review of the potential health effects of exposure to extremely low-frequency electric and magnetic fields', *Critical Reviews in Biomedical Engineering*, 47: 323-47.

Henson, R. M., A. Ortigoza, K. Martinez-Folgar, F. Baeza, W. Caiaffa, A. Vives Vergara, A. V. Diez Roux, and G. Lovasi. 2020. 'Evaluating the health effects of place-based slum upgrading physical environment interventions: A systematic review (2012–2018)', *Social Science and Medicine*, 261.

Kabisch, N., M. van den Bosch, and R. Lafortezza. 2017. 'The health benefits of nature-based solutions to urbanization challenges for children and the elderly – A systematic review', *Environmental Research*, 159: 362-73.

Katoto, P. D. M. C., L. Byamungu, A. S. Brand, J. Mokaya, H. Strijdom, N. Goswami, P. De Boever, T. S. Nawrot, and B. Nemery. 2019. 'Ambient air pollution and health in Sub-Saharan Africa: Current evidence, perspectives and a call to action', *Environmental Research*, 173: 174-88.

McCrorie, P. R. W., C. Fenton, and A. Ellaway. 2014. 'Combining GPS, GIS, and accelerometry to explore the physical activity and environment relationship in children and young people - a review', *International Journal of Behavioral Nutrition and Physical Activity*, 11.

Nordbø, E. C. A., H. Nordh, R. K. Raanaas, and G. Aamodt. 2018. 'GIS-derived measures of the built environment determinants of mental health and activity participation in childhood and adolescence: A systematic review', *Landscape and Urban Planning*, 177: 19-37.

Rocha, N. P., A. Dias, G. Santinha, M. Rodrigues, A. Queirós, and C. Rodrigues. 2019. "Smart Cities and Public Health: A Systematic Review." In, edited by M. M. Cruz-Cunha, J. E. Varajao, R. Martinho, R. Rijo, E. Peres and D. Domingos, 516-23. Elsevier B.V.

Salgado, M., J. Madureira, A. S. Mendes, A. Torres, J. P. Teixeira, and M. D. Oliveira. 2020. 'Environmental determinants of population health in urban settings. A systematic review', *BMC Public Health*, 20.

Sánchez-González, D., F. Rojo-Pérez, V. Rodríguez-Rodríguez, and G. Fernández-Mayoralas. 2020. 'Environmental and psychosocial interventions in age-friendly communities and active ageing: A systematic review', *International Journal of Environmental Research and Public Health*, 17: 1-35.

Schinasi, L. H., T. Benmarhnia, and A. J. De Roos. 2018. 'Modification of the association between high ambient temperature and health by urban microclimate indicators: A systematic review and meta-analysis', *Environmental Research*, 161: 168-80.

Singh, A., L. Daniel, E. Baker, and R. Bentley. 2019. 'Housing Disadvantage and Poor Mental Health: A Systematic Review', *American Journal of Preventive Medicine*, 57: 262-72.

Tanner, L. M., S. Moffatt, E. M. G. Milne, S. D. H. Mills, and M. White. 2013. 'Socioeconomic and behavioural risk factors for adverse winter health and social outcomes in economically developed countries: A systematic review of quantitative observational studies', *Journal of Epidemiology and Community Health*, 67: 1061-67.

Tsai, A. C. 2015. 'Home foreclosure, health, and mental health: A systematic review of individual, aggregate, and contextual associations', *PLoS ONE*, 10.

Tseng, E., A. Zhang, O. Shogbesan, K. A. Gudzune, R. F. Wilson, H. Kharrazi, L. J. Cheskin, E. B. Bass, and W. L. Bennett. 2018. 'Effectiveness of Policies and Programs to Combat Adult Obesity: a Systematic Review', *Journal of General Internal Medicine*, 33: 1990-2001.

Turley, R., R. Saith, N. Bhan, E. Rehfuess, and B. Carter. 2013. 'Slum upgrading strategies involving physical environment and infrastructure interventions and their effects on health and socio-economic outcomes', *Cochrane Database of Systematic Reviews*, 2013.

Vásquez-Vera, H., L. Palència, I. Magna, C. Mena, J. Neira, and C. Borrell. 2017. 'The threat of home eviction and its effects on health through the equity lens: A systematic review', *Social Science and Medicine*, 175: 199-208.
